# Supplemental selenium source on gut health: insights on fecal microbiome and fermentation products of growing puppies

**DOI:** 10.1093/femsec/fiaa212

**Published:** 2020-10-12

**Authors:** Ana Margarida Pereira, Carlo Pinna, Giacomo Biagi, Claudio Stefanelli, Margarida R G Maia, Elisabete Matos, Marcela A Segundo, António J M Fonseca, Ana Rita J Cabrita

**Affiliations:** LAQV, REQUIMTE, ICBAS, Instituto de Ciências Biomédicas de Abel Salazar, Universidade do Porto, Rua de Jorge Viterbo Ferreira, 228, 4050–313 Porto, Portugal; Dipartimento di Scienze Mediche Veterinarie, Università di Bologna, Via Tolara di Sopra, 43, 40064 Ozzano dell'Emilia (BO), Italy; Dipartimento di Scienze Mediche Veterinarie, Università di Bologna, Via Tolara di Sopra, 43, 40064 Ozzano dell'Emilia (BO), Italy; Dipartimento di Scienze per la Qualità della Vita, Università di Bologna, Corso d'Augusto, 237, 47921 Rimini (RN), Italy; LAQV, REQUIMTE, ICBAS, Instituto de Ciências Biomédicas de Abel Salazar, Universidade do Porto, Rua de Jorge Viterbo Ferreira, 228, 4050–313 Porto, Portugal; SORGAL, Sociedade de Óleos e Rações S.A., Estrada Nacional 109 Lugar da Pardala, 3880-728 S. João Ovar, Portugal; LAQV, REQUIMTE, Departamento de Ciências Químicas, Faculdade de Farmácia, Universidade do Porto, Rua de Jorge Viterbo Ferreira, 228, 4050–313 Porto, Portugal; LAQV, REQUIMTE, ICBAS, Instituto de Ciências Biomédicas de Abel Salazar, Universidade do Porto, Rua de Jorge Viterbo Ferreira, 228, 4050–313 Porto, Portugal; LAQV, REQUIMTE, ICBAS, Instituto de Ciências Biomédicas de Abel Salazar, Universidade do Porto, Rua de Jorge Viterbo Ferreira, 228, 4050–313 Porto, Portugal

**Keywords:** gut microbiome, selenium, nutrition, dog, age, organic minerals

## Abstract

Selenium is an essential trace element that can modulate the gut microbiome with an impact on host health. The present study aimed to evaluate the effects of organic (selenium-enriched yeast) vs inorganic (sodium selenite) selenium source on fecal end-fermentation products and gut microbiome of puppies from 20 to 52 weeks of age. Alpha and beta diversity of the gut bacterial community were affected by age but not by gender or selenium source. The relative abundance of taxa was differently affected by age, and the DNA concentration of all selected bacterial groups increased with age, although total volatile fatty acids (VFA), acetate, propionate, caproate and lactate concentrations decreased. Organic selenium was associated with a higher concentration of total VFA, propionate and butyrate, a higher number of DNA copies of *Lactobacillus*, and a trend to lower DNA copies of *Escherichia coli*. Effects on fecal microbiome during growth differed with selenium source. Females had higher fecal end-fermentation products related to protein degradation, whereas males had higher DNA concentration of *Bifidobacterium*. Organic selenium might be beneficial over inorganic for dog food supplementation due to the positive modulation of the gut microbiome observed in puppies.

## Teaser

Supplementation of dog food with organic selenium over inorganic selenium positively modulated the gut microbiome of growing puppies which might contribute to gut health.

## INTRODUCTION

The complex gut microbiome constitutes an intricate ecosystem that impacts the health of its host (Guard *et al*. 2017). Both the structure and composition of the gut microbiome are significantly affected by genetic and environmental factors. Indeed, this dynamic ecosystem undergoes modifications throughout the life of the host in response to normal changes in physiological states, such as growth and aging (Benno *et al*. 1992; Guard *et al*. 2017) or disease-induced situations (Barko *et al*. 2018). Among external factors, diet is the one that most rapidly alters the gut microbiome (Alessandri *et al*. 2019), having a positive or a negative impact on the host health and well-being.

Selenium is an essential trace element associated with antioxidant mechanisms, thyroid hormone metabolism and modulation of immune function (Roman, Jitaru and Barbante 2014). There are several mechanisms of selenium action in the gut that favor the microbiome, mostly due to its ability to reduce intestinal local inflammation, contributing to an adequate environment for the microbial community. The ability of selenium to enhance immunity is not just determined by its direct action on the host, but also through its effects in the microbiome, that will increase or decrease the susceptibility to infections provoked by specific microorganisms (Zhai *et al*. 2018). A limited number of detailed studies have evaluated the effect of selenium supplementation on the gut microbiome of fish (Kousha, Yeganeh and Amirkolaie 2019; Victor *et al*. 2019) and mammals (Kasaikina *et al*. 2011; Lv *et al*. 2015; Zhai *et al*. 2018). In summary, these studies point towards a positive impact of selenium supplementation on bacteria diversity (Victor *et al*. 2019), an increase of beneficial bacteria (Lv *et al*. 2015; Ren *et al*. 2016; Kousha, Yeganeh and Amirkolaie 2019) and a lower predisposition for infections (Zhai *et al*. 2018). Indeed, the *in vitro* study of Gangadoo *et al*. (2019) with rooster gut microbiota, showed a significant effect of selenium on the reduction of *Enterococcus cecorum*, an emerging poultry pathogen, without significant changes in the total microbial community. Similarly, an *in vivo* trial with dogs showed that dietary supplementation with a selenium/zinc enriched probiotic increased the proportions of *Lactobacillus* and *Bifidobacterium* and decreased those of *Escherichia coli, Staphylococcus* and *Enterococcus* (Ren *et al*. 2011).

The selenium requirements of animals are met through selenium sourced by raw ingredients and supplemental selenium. Inorganic sources of selenium are the most commonly used to supplement dog food, but organic selenium sources are more bioavailable for the animal (van Zelst *et al*. 2016). The canine gut microbiome has only recently begun to be studied and, to the best of our knowledge, no *in vivo* study has been performed to evaluate the effects of different sources of supplemental selenium on dogs' gut microbiome. In this context, the present study aimed to directly compare the effects of sodium selenite (inorganic selenium, SeInorg) and selenium yeast (organic selenium, SeOrg) supplemented at equal selenium levels in complete dry dog foods on the gut microbiome of puppies from 20 to 52 weeks of age. For that, fresh feces were collected at five-time points during growth, allowing us to explore the effects of selenium source, age and the interaction between selenium source and age on the bacterial profile, diversity and fecal fermentative end-products. This approach is expected to reveal possible existing interactions on the gut microbiome, rather than the evaluation of isolated genetic or environmental effects.

## MATERIALS AND METHODS

The trial was approved by the Local Animal Ethics Committee of Abel Salazar Biomedical Sciences Institute, University of Porto, and licensed by the Portuguese Directorate-General of Food and Veterinary Medicine (permit N.° 206/2017). Trained scientists in research animal care (FELASA category C) conducted the experiments, respecting good animal welfare practices.

### Animals and diets

A total of 12 Beagle puppies (6 males and 6 females) participated in the study from 12 until 52 weeks of age. The trial followed a complete randomized block design, in which puppies were distributed into six blocks of two animals and one puppy from each block was randomly allocated to one of two diets, only differing in the source of supplemental selenium. Both diets were complete dry foods formulated to meet nutrient and energy requirements of puppies after weaning up to 1-year-old (FEDIAF 2019) supplemented with either 220 μg/kg of sodium selenite (SeInorg); or with 5 mg/kg of selenium-enriched yeast from *Saccharomyces cerevisiae* (Selplex^®^, Alltech, Nicholasville, KY; SeOrg; Table 1). Regardless of the source, the amount of supplemental selenium corresponded to ca. 20% of total selenium present in diets, which covered the daily requirements of dogs. The daily food intake was calculated to meet the metabolizable energy of puppies using the equation proposed by the National Research Council (2006). Dogs were kept in the university kennel and fed their daily amount in three individual meals (9.00, 14.00 and 17.00 h) up to 22 weeks of age, and thereafter in two meals (9.00 and 17.00 h). Fresh drinking water was provided *ad libitum*. The temperature and relative humidity of the kennel were monitored daily. Food consumption was registered daily. Hemogram, serum chemistry and urinalysis were performed regularly (each month up to 28 weeks of age and every two months after the 28^th^ weeks of age) to check for dogs’ health.

**Table 1.** Ingredient (g/kg as is) and chemical composition (g/kg dry matter, unless other units are indicated) of diets.

Ingredient	Both diets	
Poultry by-product meal	203	
Broken rice	200	
Wheat gluten	100	
Pea starch	100	
Poultry fat	99	
Wheat	90	
Hydrolyzed salmon	50	
Dehulled faba beans	50	
Palatability enhancer	40	
NuPro® Yeast	30	
Apple pomace	25	
Sugar beet pulp	25	
Premix^1^	15	
Fish oil	11	
Mono-ammonium phosphate	10	
Milled salt	6	
Sodium hexametaphosphate	0.3	
Chemical composition	Diet SeInorg	Diet SeOrg
Dry matter	917	932
Ash	62.1	61.8
Crude protein, g	333	333
Starch	323	322
Neutral detergent fiber	118	123
Acid detergent fiber	23.0	22.5
Acid detergent lignin	20.8	21.8
Gross energy (MJ)	21.1	21.2
Selenium (μg)	564	567

1 Premix per kg of diet: vitamin A 14 950 UI; vitamin D_3_ 1560 UI; vitamin E 98.0 mg; thiamine 2 mg; riboflavin 4 mg, niacin 30 μg; cobalamin 30 μg; vitamin B_6_ 3 mg; folic acid 495 μg; biotin 150 μg; vitamin K 2 mg; pantothenic acid 20 mg; CuSO_4_ 8 mg; KI 2 mg; MnSO_4_ 5 mg; ZnSO_4_ 100 mg: Selenium: SeInorg contains 220 μg of Na_2_SeO_3_ and SeOrg contains 5 mg of Selplex^®^.

### Sample collection and storage

In the last two days of 20, 28, 36, 44 and 52 weeks of age, fresh feces were collected within 1 h of defecation. Subsequently, fecal samples were pooled, weighed and split to be frozen at −80°C for fecal microbiota analysis and −20°C for the remaining analyses.

### Ammonia-N, pH and biogenic amines

The determination of ammonia-N followed the protocol proposed by Valente *et al*. (2017). Briefly, 1 g of feces was diluted in 200 mL of ultrapure water (18.2 MΩ cm; Sartorius Arium^®^, Goettingen, Germany) and subjected to gas-diffusion microextraction with *o*-phthalaldehyde labeling for fluorimetric determination in a microplate reader (Synergy HT, Bio-Tek Instruments, Bad Friedrichshall, Germany). The pH of feces was measured in feces diluted to 1:10 in water using a potentiometer (pH and Ion-Meter GLP 22, Crison, Barcelona, Spain). For the determination of biogenic amines, 1 g of feces was diluted in 4 mL of 0.3 M perchloric acid and analyzed by high-performance liquid chromatography coupled to a fluorescence detector as described by Stefanelli, Carat and Rossoni (1986).

### Lactate and volatile fatty acids

Lactate was determined using a commercial kit (d- / l-Lactic acid, Nzytech, Lisboa, Portugal) adapted to a microplate format to allow UV detection in a microplate reader (Synergy HT, Bio-Tek Instruments, Winooski, VT). The sample preparation included solubilization of 1 g of feces into 10 mL of ultrapure water aided by vortex and ultrasound (5 min). The samples were centrifuged for 15 min at 2415 × *g*, at 4°C. The supernatant was recovered, filtered using a 0.45 µm pore size polyethersulfone syringe filter (VWR International, Amadora Portugal), and assayed with the commercial kit. Lactic acid is presented as the sum of d- and l-lactic acid forms.

The concentration of VFA was determined by gas chromatography using a Shimadzu GC-2010 Plus (Shimadzu Corporation, Kyoto, Japan) equipped with a capillary column (HP-FFAP, 30 m  ×  0.25 mm  ×  0.25 μm; Agilent Technologies, Santa Clara, CA) and a flame ionization detector. For sample preparation, 1 g of feces was solubilized in 10 mL of 25% ortho-phosphoric acid solution with an internal standard (4 mM 3-methyl valerate, Sigma Aldrich, St. Louis, MO) and centrifuged for 60 min at 5251 × *g* at 4°C. The supernatant was filtered using a 0.45 µm pore size polyethersulfone syringe filters (VWR International) and injected for analysis. Individual VFA were identified by comparison of retention times with a commercial standard and quantified with the internal standard method as described by Maia *et al*. (2016).

### DNA isolation and 16S rRNA gene amplicon sequencing

Bacterial genomic DNA was extracted from 200 mg of frozen fecal samples. The DNA extraction was performed using a stool DNA isolation kit (Norgen Biotek Corp., ON, Canada) following all the procedures recommended by the manufacturer. The purity and concentration of the isolated DNA were evaluated with a spectrophotometer (DS-11, DeNonix^®^, Wilmington, DE). The DNA template was diluted to 50 ng/μL and stored at −20°C for further analysis.

The hypervariable V3–V4 regions of the 16S rRNA encoding gene were sequenced at StarSEQ (Mainz, Germany). The F341/R806b primer set and AccuStart II PCR ToughMix^®^ (Quantabio, Beverly, MA) were used for the reaction as described by Takahashi *et al*. (2014), and Apprill *et al*. (2015). Amplicons were generated by a single-step of 33 cycles using a Thermocycler T-Professional (Biometra, Göttingen, Germany) and checked for quality with QIAxcel^®^ capillary electrophoresis (Qiagen, Hilden, Germany), normalized and pooled for quantification. Over 15% of the PhiX control library was spiked into the amplicon pool to improve the unbalanced and biased base composition. The sequencing primers for forward sense strand (5′-GGCTGACTGACT-3′) and reverse sense strand (5′-CCAATTACCATA-3′) were added to MiSeq Reagent Kit V3 (Illumina, San Diego, CA) and positive control (ZymoBIOMICS Microbial Community DNA Standard; Zymo Research Corp., CA). The 2 × 300 bp pair-end sequencing was run on a MiSeq platform (Illumina, San Diego, CA).

### Sequencing analysis

The sequences from the MiSeq Illumina were analyzed using the QIIME 2 version 2018.6 (Bolyen *et al*. 2019). Raw reads were de-multiplexed and quality checked by FastQC (Andrews 2010). Paired-end reads were joined by the tool PEAR. Low-quality reads were removed. Reads were corrected, chimeras were removed and Amplicon Sequence Variants (ASVs) were obtained by the deblur workflow (Amir *et al*. 2017). Then, a multiple sequence alignment (Katoh *et al*. 2002) and a phylogenetic tree were generated (Price, Dehal and Arkin 2009). Alpha diversity rarefaction curves were generated for each category (selenium source, gender and the week of age) and each sample individually. Taxonomy was assigned to ASVs using a Naive–Bayes approach of the scikit-learn Python library (Bokulich *et al*. 2018) and the SILVA database (Quast *et al*. 2013). Interactive stacked bar-charts of the taxonomic abundances of each category and each sample were generated. Alpha and beta diversity metrics were calculated after normalization by rarefaction (at the lowest sample size). Alpha diversity metrics were calculated using Shannon's diversity index and Faith's phylogenetic diversity to assess the community's richness and Pielou's Evenness to assess the community's evenness. Beta diversity metrics calculated were Weighted and Unweighted UniFrac distances to assess community dissimilarity. The Principal Coordinate Analysis was used to plot the distance matrixes.

### Quantitative real-time PCR

Total bacteria, *Lactobacillus* spp., *Enterococcus* spp., *Bifidobacterium* spp., *E. coli, F. prausnitzii* and *Clostridium* cluster I were quantified by quantitative polimerase chain reaction (qPCR). Detailed information of qPCR assay is presented in Table 2. Amplification was run in duplicate with a total volume of 15 μL, 1.5 μL of DNA template, 7.5 μL of 2x SensiFASTNo-ROX PCRMasterMix (Bioline GmbH, Luckenwalde, Germany), 4.8 μL of nuclease-free water and 0.6 μL of each 10 pmol primers. Amplification and detection were carried out in a CFX96 Touch thermal cycler (Bio-Rad, Hercules, CA) after an initial denaturation of 2 min at 95°C, followed by 40 cycles of 95°C for 5 s, primer annealing (56–64°C) for 10 s and 72°C for 8 s. The standard curves were obtained from seven 10-fold dilution series of the target species genomic DNA. Standard curves were run in triplicates. A negative control (DNase-free water) was run for each primer assay. Melting curve analysis was performed after the amplification to check the consistency of the amplification of the single product with the set melting temperature.

**Table 2.** Primers used in the qPCR assay.

Target species	Primer	Sequence (5′→3′)	Annealing temperature (°C)	Reference
Total bacteria (194 bp)	UniF	CCTACGGGAGGCAGCAG	62	Muyzer de Waal and Uitterlinden (1993)
	UniR	ATTACCGCGGCTGCTGG		
*Clostridium* cluster I (231 bp)	CI-F1	TACCHRAGGAGGAAGCCA	59	Song, Liu and Finegold (2004)
	CI-R2	GTTCTTCCTAATCTCTACGCAT		
*Lactobacillus* spp. (341 bp)	LacF	AGCAGTAGGGAATCTTCCA	64	Malinen *et al*. (2005)
	LacR	CACCGCTACACATGGAG		
*Bifidobacterium* spp. (243 bp)	BifF	TCGCGTCYGGTGTGAAAG	56	Rinttila *et al*. (2004)
	BifR	CCACATCCAGCRTCCAC		
*Escherichia coli* (340 bp)	*E. coli* Fw	GTTAATACCTTTGCTCATTGA	59	Malinen *et al*. (2003)
	*E. coli* Rv	ACCAGGGTATCTAATCCTGTT		
*Faecalibacterium prausnitzii* (140 bp)	Fprau 07	CCATGAATTGCCTTCAAAACTGTT	59	Sokol *et al*. (2009)
	Fprau 02	GAGCCTCAGCGTCAGTTGGT		
*Enterococcus* spp. (144 bp)	EnteroF	CCCTTATTGTTAGTTGCCATCATT	59	Rinttila *et al*. (2004)
	EnteroR	ACTCGTTGTACTTCCCATTGT		

### Statistical analysis

QIIME 2 workflow was used to select ASV, sequence alignment, inferring phylogenetic trees and phylogenetic and taxon-based analysis of alpha and beta diversity within and between samples as described by Caporaso *et al*. (2010). Given the nonparametric nature of microbiota data, indices of alpha diversity data were analyzed using the Kruskal–Wallis test. Differences in beta diversity were analyzed using the non-parametric Permutational multivariate analysis of variance (PERMANOVA) with 999 permutations. *P*-values were corrected using the Benjamini–Hochberg False Discovery Rate method.

DNA concentration, fecal pH and concentration of end-fermentation products were analyzed according to a Generalized Linear Mixed Model with repeated measurements (SAS^®^ University Edition 2019, Cary, NC). The model included selenium source, gender, age and selenium source × age interaction (if the interaction had *P* < 0.1) as fixed effects, block as a random effect and age in the subject dog as a repeated measure. Taxa with relative abundance > 0.01% and present in at least 50% of the samples (which corresponded to 95% of total reads) was analyzed using a Negative Binomial Generalized Linear Mixed Model (Proc Glimmix of SAS^®^) including selenium source, gender, age and selenium source × age interaction (if the interaction had *P* < 0.1) as fixed effects, and block as a random effect. The statistical level of significance was considered for *P* < 0.05, while the trend was set for *P* < 0.1. The least significant difference post-hoc test was used to compare means.

## RESULTS

### Sequencing analysis and alpha diversity

The total number of sequences obtained after filtering for quality, trimming length and assigning taxonomy was 5151 331 from 60 samples with an average of 85 885 ± 24 931 reads per sample (range 35 646–137 908). A total of 1 886 operational taxonomy units (OTU) were identified, and 174 assigned to the genus level. After rarefaction, normalizing to the sample with the lowest number of sequences (5782), 341 138 sequences were retained (46.1%) in 58 samples. The number of observed OTUs per sample ranged from 65 to 171 (Table 3). Rarefaction curves were adequate for the analysis, as they all tended to a plateau (Figures S1–S3, Supporting Information). The number of OTUs and richness community indexes (Shannon's diversity index and Faith's phylogenetic diversity), and community evenness (Pielou's Evenness) were affected by the age of puppies (*P* < 0.001; Tables 3 and S1, Supporting Information), with alpha diversity lower at 20 weeks of age, but they were not affected by selenium source nor gender (*P* > 0.05, Tables 3, S2 and S3, Supporting Information).

**Table 3.** Total number of reads per sample assigned to OTUs and alpha-metrics (mean ± standard deviation), namely Shannon's diversity index, Faith's phylogenetic diversity and Pielou's Evenness in the communities of fresh feces from puppies fed the inorganic (SeInorg) and the organic (SeOrg) selenium supplemented diets, collected at 5-time points from 20 to 52 weeks of age.

Categories	n	Reads	OTUs	Shannon's diversity index	Faith's phylogenetic diversity	Pielou's evenness
Diet						
SeInorg	28	88 128 ± 26 512	132 ± 23	4.52 ± 0.749	10.8 ± 1.32	0.64 ± 0.089
SeOrg	30	83 583 ± 23 473	126 ± 23	4.38 ± 0.881	10.4 ± 1.23	0.62 ± 0.107
Weeks of age						
20	12	90 391 ± 13 261	103 ± 27	3.33 ± 0.716^b^	9.19 ± 1.502^b^	0.50 ± 0.093^b^
28	12	90 280 ± 13 083	138 ± 13	4.72 ± 0.458^a^	11.2 ± 0.80^a^	0.66 ± 0.056^a^
36	12	96 859 ± 14 206	138 ± 13	4.70 ± 0.568^a^	11.1 ± 0.72^a^	0.66 ± 0.071^a^
44	12	101 861 ± 28 237	142 ± 19	4.96 ± 0.612^a^	11.2 ± 0.81^a^	0.69 ± 0.072^a^
52	10	49 887 ± 11 004	124 ±15	4.54 ± 0.519^a^	10.2 ± 1.07^b^	0.65 ± 0.060^a^
Gender						
F	29	85 087 ± 25 480	128 ± 24	4.42 ± 0.878	10.5 ± 1.40	0.63 ± 0.107
M	29	86 623 ± 24 782	130 ± 23	4.48 ± 0.763	10.7 ± 1.14	0.64 ± 0.091

a–b Values in the same column that share a common superscript are not statistically different (*P* > 0.05).

Letters from gender designate: F: female; M: male.

### Beta diversity

**Figure 1.**
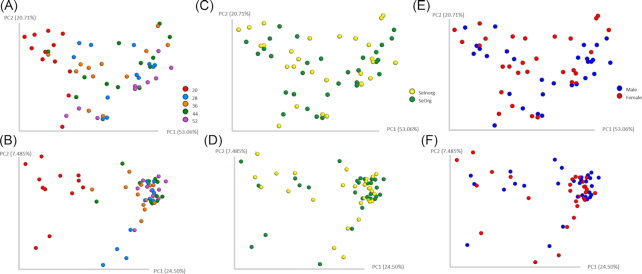
Beta diversity metrics. Principal coordinate analysis of Weighted (**A, C** and **E**) and Unweighted (**B, D** and **F**) UniFrac distances of samples showing the effect of weeks of age (A and B) selenium source (C and D) and gender (E and F) of puppies.

Principal coordinate analysis based on Weighted and Unweighted UniFrac distances showed differences associated with age (Fig. 1A and B). Samples collected at 20 weeks of age differed from all other weeks, suggesting changes in the overall microbiome composition (Fig. 1A) and higher weight of low-abundance taxa (Fig. 1B). Though, no quantitative and qualitative differences in microbiota diversity were observed between males and females nor between SeInorg and SeOrg (Fig. 1C–F). Differences among weeks were confirmed by PERMANOVA analysis on Unweighted UniFrac distances (pseudo-F = 5.58; *P* = 0.001¸ pairwise PERMANOVA results available in Table S4, Supporting Information), which also revealed no effects of selenium source (pseudo-F = 0.70; *P* > 0.835) and gender (pseudo-F = 0.71; *P* > 0.843). Similarly, PERMANOVA analysis on Weighted UniFrac distances showed an effect of age (pseudo-F = 6.99; *P* = 0.001; pairwise PERMANOVA results available in Table S4, Supporting Information), but not of selenium source (pseudo-F = 0.35; *P* > 0.814) and gender (pseudo-F = 1.14; *P* > 0.332).

### Microbiome profiling

**Figure 2.**
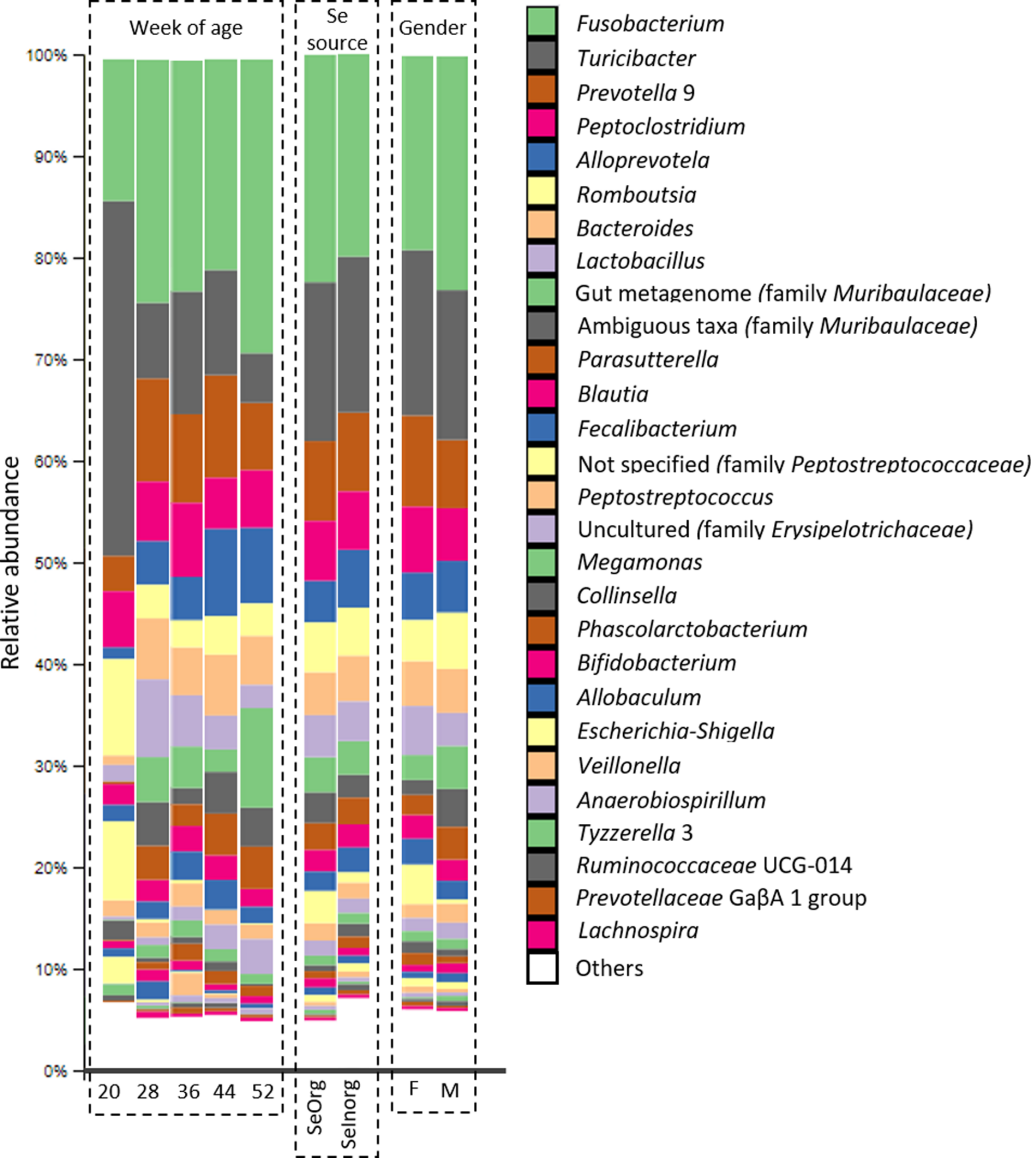
Relative abundance (%) of genera in samples according to weeks of age, selenium source and gender of puppies. Genera with relative abundance < 0.5% were pooled and named ‘Others’.

After normalization of sequence reads into relative abundances, 10 phyla, 15 classes, 33 orders, 62 families and 174 genera were identified. From these, only 5 phyla, 9 classes, 11 order and 17 families presented relative abundances above 1%, and 28 genera above 0.5% (Fig. 2). *Fusobacterium, Turicibacter, Prevotella* 9 and *Peptoclostridium* represented together roughly 50% of the total genus presented, whereas the sum of 146 genera with relative abundances lower than 0.05%, corresponded to 5–10%.

We have further investigated the effects of age, selenium source, gender and interaction between selenium source and age on the relative abundance of specific taxa using a negative binomial generalized linear mixed model, to remove the variation from the effects (Table 4).

**Table 4.** Most abundant taxa in fresh feces of puppies from 20 to 52 weeks of age fed the inorganic (SeInorg) and the organic (SeOrg) selenium supplemented diets.

	Age (weeks)							Selenium source				Gender			
Taxa	20	28	36	44	52	sd	*P* value	SeInorg	SeOrg	sd	*P* value	F	M	sd	*P* value
p_Actinobacteria*	5.87^a^	5.56^a,b^	5.28^b^	5.54^a,b^	4.37^c^	0.177	<0.001	5.36	5.28	0.138	0.635	5.23	5.41	0.144	0.366
p_Bacteroidetes	6.81^c^	8.23^a^	7.92^a,b^	8.40^a^	7.75^b^	0.170	<0.001	7.85	7.79	0.108	0.731	7.74	7.90	0.108	0.311
p_Epsilonbacteraeota	1.95^b^	3.39^a^	3.11^a^	2.70^a,b^	1.93^b^	0.373	0.009	2.62	2.61	0.296	0.970	2.69	2.55	0.301	0.746
p_Firmicutes^†^	9.30^a^	8.51^b^	8.57^b^	8.65^b^	7.85^c^	0.109	<0.001	8.56	8.59	0.069	0.768	8.66	8.49	0.069	0.085
p_Proteobacteria	6.14	6.28	6.00	6.57	5.90	0.215	0.132	6.40	5.95	0.185	0.103	6.09	6.26	0.180	0.502
c_Actinobacteria	4.68	5.02	4.68	4.43	3.98	0.272	0.124	4.41	4.70	0.173	0.261	6.01	5.92	0.303	0.848
c_Alphaproteobacteria	4.58^a^	2.21^b^	2.22^b^	2.26^b^	−1.03^c^	0.472	<0.001	2.21	1.88	0.299	0.446	4.75	4.70	0.105	0.761
c_Bacilli	5.55^b,c^	6.84^a^	6.34^a,b^	6.03^a,b^	5.07^c^	0.398	0.008	6.05	5.88	0.309	0.714	6.05	6.17	0.218	0.705
c_Coriobacteriia**	5.60^a^	4.84^b^	4.66^b^	5.08^b^	3.46^c^	0.165	<0.001	4.89	4.56	0.104	0.033	7.91	7.78	0.071	0.198
c_Gammaproteobacteria	5.81	6.26	5.98	6.56	5.94	0.2636	0.183	6.35	5.87	0.225	0.147	5.60	5.37	0.171	0.344
o_Clostridiales^†^	8.49^a^	7.78^b^	7.80^b^	7.96^b^	7.21^c^	0.112	<0.001	7.85	7.85	0.071	0.971	4.39	4.72	0.172	0.194
o_Selenomonadales	4.45^d^	5.53^b,c^	6.38^a^	6.02^a,b^	5.04^dc^	0.269	<0.001	5.72	5.25	0.170	0.059	2.15	1.94	0.297	0.629
f_Burkholderiaceae	4.35^c^	6.09^a,b^	5.66^b^	6.43^a^	5.71^b^	0.240	<0.001	5.67	5.63	0.180	0.882	5.49	5.80	0.161	0.181
f_Eggerthellaceae*	2.33^a^	2.62^a^	2.25^a^	2.48^a^	1.02^b^	0.284	0.005	2.09	2.19	0.204	0.733	1.86	2.42	0.211	0.090
f_Enterobacteriaceae	6.07^a^	3.39^b^	2.76^b^	2.11^b^	0.40^c^	0.476	<0.001	2.74	3.15	0.306	0.377	2.74	3.15	0.306	0.361
f_Erysipelotrichaceae	8.55^a^	7.08^b^	7.23^b^	7.43^b^	6.40^c^	0.207	<0.001	7.19	7.49	0.196	0.304	7.32	7.36	0.183	0.893
f_Lachnospiraceae*	6.36^a^	6.39^a^	6.25^a^	6.42^a^	5.43^b^	0.127	<0.001	6.19	6.15	0.080	0.747	6.15	6.19	0.080	0.738
f_Muribaculaceae	3.23^b^	6.89^a^	6.25^a^	6.59^a^	6.80^a^	0.340	<0.001	5.95	5.96	0.265	0.997	5.51	6.40	0.267	0.012
f_Peptostreptococcaceae	8.18^a^	7.26^b^	7.26^b^	7.29^b^	6.60^c^	0.169	<0.001	7.28	7.36	0.107	0.609	7.39	7.25	0.107	0.377
f_Rhizobiaceae	4.37^a^	2.00^b^	2.04^b^	2.03^b^	−1.04^c^	0.465	<0.001	2.04	1.72	0.296	0.445	1.97	1.79	0.292	0.671
f_Ruminococcaceae	6.33^a^	5.76^b^	6.07^a,b^	6.43^a^	5.15^c^	0.160	<0.001	6.11	5.78	0.110	0.040	5.95	5.95	0.113	0.983
f_Succinivibrionaceae	2.29^b^	4.09^a^	4.70^a^	4.31^a^	3.79^a^	0.328	<0.001	3.70	3.97	0.211	0.396	4.07	3.60	0.209	0.127
f_Veillonellaceae	4.26^c^	5.09^b,c^	6.06^a^	5.47^a,b^	4.35^c^	0.297	<0.001	5.27	4.82	0.188	0.099	5.12	4.97	0.190	0.588
g_Allisonella*	0.67^c^	2.04^a,b^	1.46^b,c^	2.47^a^	1.22^b,c^	0.394	0.034	1.98	1.16	0.249	0.027	1.79	1.36	0.253	0.242
g_*Allobaculum*	4.47^a,b^	5.27^a^	2.86^c^	3.63^b,c^	3.43^b,c^	0.384	<0.001	3.89	3.97	0.292	0.827	3.68	4.19	0.289	0.189
g_*Alloprevotella*	5.18^c^	6.32^b^	6.18^b^	7.06^a^	6.29^b^	0.220	<0.001	6.30	6.11	0.139	0.325	6.18	6.23	0.140	0.812
g_*Allorhizobium-Neorhizobium-*															
*-Pararhizobium-Rhizobium*	5.49^c^	6.91^a,b^	6.66^a,b^	7.24^a^	6.52^b^	0.228	<0.001	6.58	6.55	0.144	0.886	6.45	6.68	0.145	0.265
g_*Anaerobiospirillum*	2.27^b^	3.66^a^	4.33^a^	4.20^a^	3.65^a^	0.345	0.002	3.46	3.78	0.224	0.345	3.79	3.45	0.219	0.283
g_*Anaerofilum*	1.22^b^	1.13^b^	1.69^a,b^	2.45^a^	1.39^a,b^	0.362	0.081	1.63	1.52	0.228	0.733	1.43	1.72	0.233	0.398
g_*Angelakisella*	4.17^a^	1.30^c^	1.83^b,c^	2.80^b^	1.34^c^	0.346	<0.001	2.41	2.17	0.220	0.460	2.30	2.27	0.225	0.922
g_*Bacteroides*	4.94^c^	6.64^a,b^	6.29^a^	6.75^a^	5.84^b^	0.233	<0.001	6.07	6.12	0.148	0.824	6.09	6.10	0.148	0.953
g_*Bifidobacterium*	4.64	5.03	4.68	4.42	3.98	0.288	0.169	4.40	4.69	0.183	0.278	4.38	4.71	0.182	0.207
g_*Blautia*^†^	5.73^a^	5.59^a^	5.66^a^	5.83^a^	4.74^b^	0.172	0.001	5.50	5.52	0.109	0.883	5.58	5.44	0.110	0.402
g_*Butyricicoccus*	3.84^a^	1.97^b,c^	1.86^c^	2.50^b^	0.93^d^	0.203	<0.001	2.33	2.12	0.129	0.254	2.18	2.26	0.129	0.648
g_*Candidatus* Arthromitus	3.05^a^	2.18^a,b^	2.71^a^	1.85^a,b^	0.94^b^	0.483	0.036	2.32	1.97	0.314	0.464	2.37	1.92	0.307	0.313
g_*Catenibacterium*	4.49^a^	2.38^b^	2.48^b^	1.47^b^	−1.43^c^	0.465	<0.001	2.45	1.31	0.321	0.015	2.22	1.54	0.316	0.125
g_*Clostridium sensu stricto* 1	−1.45^c^	3.06^b^	3.31^b^	3.70^a,b^	4.42^a^	0.435	<0.001	2.87	2.35	0.279	0.186	2.54	2.68	0.283	0.722
g_Collinsella*	5.53^a^	3.99^c^	4.35^b,c^	4.66^b^	2.66^d^	0.205	<0.001	4.46	4.01	0.145	0.043	4.35	4.12	0.141	0.263
g_Dubosiella*	2.19^b^	3.26^a^	3.23^a^	1.62^b^	1.22^b^	0.397	0.001	1.93	2.69	0.288	0.048	2.09	2.52	0.289	0.269
g_*Erysipelatoclostridium*	4.37^a^	3.34^b,c^	2.88^c,d^	3.64^b^	2.42^d^	0.205	<0.001	3.44	3.22	0.133	0.268	3.27	3.39	0.145	0.567
g_*Escherichia-Shigella*	6.07^a^	3.39^b^	2.75^b^	2.11^b^	0.40^c^	0.480	<0.001	2.74	3.14	0.308	0.390	2.73	3.15	0.308	0.359
g_*Faecalibacterium*	5.47^a,b^	5.33^b,c^	5.75^a,b^	5.96^a^	4.77^c^	0.208	0.002	5.53	5.38	0.148	0.507	5.55	5.36	0.149	0.386
g_*Fournierella*	0.61^c^	2.40^a,b^	2.63^a,b^	3.06^a^	2.01^b^	0.307	<0.001	2.27	2.01	0.233	0.415	2.31	1.97	0.234	0.310
g_*Fusicatenibacter*	−0.48^b^	1.50^a^	1.58^a^	1.87^a^	1.27^a^	0.293	0.001	1.16	1.14	0.186	0.958	1.63	0.67	0.184	<0.001
g_*Fusobacterium*	7.41	7.91	7.84	7.92	7.59	0.251	0.496	7.74	7.73	0.190	0.975	7.60	7.87	0.188	0.301
g_*Helicobacter*	1.90^b^	3.39^a^	3.10^a^	2.68^a,b^	1.89^b^	0.370	0.006	2.62	2.56	0.294	0.899	2.67	2.51	0.299	0.707
g_*Holdemanella*	4.03^a^	2.77^b^	2.93^b^	3.05^b^	1.92^c^	0.201	<0.001	3.16	2.72	0.127	0.017	3.05	2.83	0.128	0.219
g_*Howardella*	−0.52^c^	1.19^a,b^	0.81^a,b,c^	1.65^a^	0.10^b,c^	0.437	0.012	0.90	0.39	0.277	0.205	0.46	0.83	0.278	0.359
g_*Lachnoclostridium*	0.47^c^	3.20^a^	3.00^a^	3.25^a^	2.02^b^	0.279	<0.001	2.51	2.27	0.196	0.341	2.44	2.34	0.210	0.736
g_*Lachnospira*	−0.44^c^	4.39^a^	3.44^a,b^	3.96^a^	2.89^b^	0.426	<0.001	3.22	2.48	0.334	0.140	2.58	3.11	0.336	0.286
g_*Lachnospiraceae* NK4A136 group	1.19^b^	2.04^a,b^	2.08^a,b^	2.91^a^	1.35^b^	0.353	0.009	1.96	1.86	0.223	0.739	1.77	2.06	0.226	0.378
g_*Lactobacillus*	5.38^b,c^	6.84^a^	6.30^a,b^	6.05^a,b,c^	5.06^c^	0.418	0.012	6.05	5.80	0.315	0.573	5.98	5.87	0.309	0.800
g_*Megamonas*	2.30^c^	5.06^a^	5.22^a^	5.14^a^	4.22^b^	0.265	<0.001	4.33	4.45	0.169	0.643	4.34	4.44	0.168	0.694
g_*Negativibacillus*	0.51^c^	3.13^a,b^	2.00^a,b^	3.17^a^	1.70^b,c^	0.512	0.003	2.23	1.98	0.384	0.669	1.68	2.52	0.383	0.143
g_*Parasutterella*	2.72^c^	6.00^a,b^	5.37^b^	6.30^a^	5.69^a,b^	0.394	<0.001	5.18	5.25	0.311	0.889	4.99	5.44	0.294	0.264
g_*Phascolarctobacterium*	2.78^b^	4.48^a^	5.14^a^	5.13^a^	4.34^a^	0.363	<0.001	4.57	4.17	0.230	0.228	4.53	4.211	0.232	0.341
g_*Peptoclostridium*	6.70	6.63	6.70	6.58	5.96	0.227	0.135	6.48	6.55	0.145	0.724	6.61	6.42	0.145	0.342
g_*Peptococcus*	1.66^c^	3.08^a,b^	2.60^a,b^	3.22^a^	2.36^b,c^	0.280	0.001	2.64	2.53	0.212	0.730	2.33	2.84	0.198	0.061
g_*Peptostreptococcus*	4.79	4.74	5.25	5.00	4.57	0.406	0.690	5.00	4.73	0.341	0.563	4.52	5.21	0.335	0.133
g_*Prevotella* 9	6.26^b^	7.16^a^	6.89^a,b^	7.26^a^	6.17^b^	0.255	0.009	6.73	6.76	0.163	0.895	6.78	6.71	0.165	0.771
g_*Prevotellaceae* Ga6A1 group	2.86	3.52	3.83	3.71	2.89	0.384	0.135	3.71	3.01	0.305	0.100	3.54	3.18	0.305	0.392
g_*Romboutsia*	7.00^a^	5.78^b,c^	5.59^c^	6.29^b^	5.47^c^	0.274	0.000	5.78	6.28	0.262	0.197	5.81	6.24	0.258	0.259
g_*Ruminococcaceae* UCG-005	3.02^a,b,c^	3.14^a,b^	2.10^c^	3.87^a^	2.69^b,c^	0.377	0.015	3.30	2.63	0.295	0.132	2.66	3.27	0.285	0.141
g_*Ruminococcaceae* UCG-014	4.28^a,b^	3.58^b,c^	3.76^b,c^	4.68^a^	3.12^c^	0.306	0.008	4.43	3.34	0.197	<0.001	3.63	4.14	0.195	0.079
g_*Sutterella*	1.02^b^	3.02^a^	3.97^a^	3.93^a^	2.77^a^	0.428	0.000	2.97	2.91	0.278	0.874	3.21	2.68	0.276	0.193
g_*Turicibacter*	8.46^a^	6.58^b^	7.03^b^	7.11^b^	5.73^c^	0.283	<0.001	6.76	7.20	0.255	0.247	7.06	6.90	0.243	0.635
g_*Tyzzerella* 3	5.36^a^	3.58^b^	2.53^b,c^	1.23^c,d^	−0.37^d^	0.572	<0.001	2.49	2.44	0.357	0.932	2.75	2.17	0.378	0.325
g_[*Eubacterium*] brachy group	1.27^c^	3.55^a^	2.88^a,b^	3.67^a^	2.56^a,b^	0.307	<0.001	2.98	2.60	0.222	0.258	2.48	3.09	0.206	0.040
g_[*Ruminococcus*] *gauvreauii* group^†^	2.42^a^	2.18^a^	2.04^a^	2.43^a^	1.25^b^	0.216	0.006	2.16	1.96	0.137	0.311	2.13	1.99	0.137	0.477
g_[*Ruminococcus*] *gnavus* group*	3.05^b^	3.65^a^	3.34^a,b^	3.22^b^	2.04^c^	0.156	<0.001	3.13	2.99	0.123	0.378	2.99	3.12	0.121	0.408
g_[*Ruminococcus*] *torques* group*	2.48^c^	3.63^a,b^	3.59^a,b^	3.93^a^	3.26^b^	0.186	<0.001	3.61	3.15	0.156	0.051	3.31	3.45	0.148	0.498

a–d Values in the same row that share a common superscript are not statistically different (*P* > 0.05).

sd: standard deviation.

Letters from gender designate: F: female; M: male.

Letters before bacterial groups designate taxa: p_: phylum; c_: class; o_: order; f_: family; g_: genus.

**Interaction between selenium source and age was highly statistically significant (*P* < 0.001), *interaction between selenium source and age was statistically significant (*P* < 0.05), ^†^interaction between selenium source and age tended to be significant (*P* < 0.1).

The lowest abundance of Phylum Actinobacteria was observed at week 52 of age (*P* < 0.05), reflecting the Eggerthellaceae family.

Phylum Bacteroidetes was lower at week 20 of age (*P* < 0.001), reflecting family Muribaculaceae, and genera *Alloprevetella* and *Bacteroides* (*P* < 0.05). Conversely, phylum Firmicutes abundance was higher at 20 weeks of age and lower at 52 weeks of age (*P* < 0.001), mirroring order Clostridiales, families Erysipelotrichaceae, Peptostreptococcaceae and Ruminococcaceae. However, differences occurred at genera level of these families with *Fournierella, Fusicatenibacter, Lachnoclostridium* and *Lachnospira* relative abundance being markedly lower at week 20 (*P* < 0.05) whereas no clear pattern was observed for *Anaerofilum, Howardella, Lachnospiraceae* NK4A136 group *Negativibacillus, Peptococcus, Ruminococcaceae* UCG-005 and *Ruminococcaceae* UCG-014 among weeks (*P* < 0.05). Similarly, *Lactobacillus* (order Lactobacillales) and order Selenomonadales (family Veillonellaceae) fluctuated among weeks (*P* < 0.05), whereas genera *Phascolarctobacterium* (order Selenomonadales) was lowest at 20 weeks of age and *Clostridium sensu stricto* 1 (family Clostridiaceae 1) increased with age (*P* < 0.05). Phylum Epsilonbacteraeota fluctuated with age reflecting genus *Helicobacter* (*P* < 0.001). *Fusobacterium* was not affected by age (*P* > 0.05). Class Alphaproteobacteria decreased from 20 to 52 weeks of age (*P* < 0.001), although phylum Proteobacteria was not affected by age (P > 0.05). Class Gammaproteobacteria was not affected by age (*P* > 0,05), but family Succinivibrionaceae (also its genus *Anaerobiospirillum*) and genus *Suterella* were lowest at 20 weeks of age and similar among the other weeks, whereas genus *Parasutterella* fluctuated among weeks (*P* < 0.01), and family Enterobacteriaceae (also its genus *Escherichia-Shigella*) decreased from 20 to 52 weeks of age (*P* < 0.001).

Inorganic selenium promoted the enrichment of family Ruminococcaceae, genera *Catenibacterium, Holdemanella and Ruminococcaceae* UCG-014, (*P* < 0.05) and tended to promote the enrichment of order Selenomonadales, family Veillonellaceae and genus [*Ruminococcus*] *torques* group (*P* < 0.1).

The interaction between selenium source and age affected phylum Actinobacteria, class Coriobacteriia, families Eggerthellaceae and Lachnospiraceae, genera *Allisonella, Collinsella, Dubosiella*, [*Ruminococcus*] *gnavus* group and [*Ruminococcus*] *torques* group (*P* < 0.05, Table 5), and tended to affect phylum Firmicutes, order Clostridiales, genera *Blautia* and [*Ruminococcus*] *gauvreauii* group (*P* < 0.1, data not shown). Overall, the relative abundance of phylum Actinobacteria, class Coreobacteriia, families Eggerthellaceae and Lachnospiraceae, and genus *Dubosiella* was higher at 52 weeks of age of puppies fed SeOrg diet.

**Table 5.** Most abundant taxa in fresh feces from puppies as affected (*P* < 0.05) by the interaction between selenium source (inorganic, SeInorg; organic, SeOrg) and age (20–52 weeks).

	SeInorg					SeOrg						
Taxa	20	28	36	44	52	20	28	36	44	52	sd	*P*-value
p_Actinobacteria	6.15^a^	5.81^b,c^	5.33^b,c^	5.75^a,b^	3.78^d^	5.58^a,b,c^	5.30^a,b^	5.22^b,c^	5.33^b,c^	4.95^c^	0.244	0.002
c_Coriobacteriia	6.02^a^	5.22^b,c^	4.74^c,d^	5.57^a,b^	2.90^f^	5.18^b,c^	4.45^d,e^	4.58^c,d,e^	4.58^c,d,e^	4.03^e^	0.233	<0.001
f_Eggerthellaceae	2.51^a^	2.97^a^	2.44^a^	2.49^a^	0.03^a^	2.15^a^	2.26^a^	2.05^a^	2.48^a^	2.01^b^	0.402	0.027
f_Lachnospiraceae	6.22^a^	6.57^a^	6.37^a^	6.61^a^	5.17^c^	6.50^a^	6.22^a^	6.13^a,b^	6.23^a^	5.69^b^	0.179	0.049
g_*Allisonella*	2.50^a,b^	1.90^a,b,c^	1.24^b,c^	2.73^a^	1.51^a,b,c^	−1.17^d^	2.19^a,b,c^	1.68^a,b,c^	2.21^a^	0.93^c^	0.551	0.021
g_*Collinsella*	5.96^a^	4.22^b,c^	4.44^b,c^	5.37^a^	2.33^d^	5.10^a,b^	3.75^dc^	4.27^b,c^	3.95^c^	2.98^d,e^	0.294	0.013
g_*Dubosiella*	2.54^a,b,c^	3.13^a,b^	3.11^a,b^	1.08^a,b,c^	−0.23^d^	1.85^b^c	3.39^a^	3.35^a^	2.17^dc^	2.67^a,b,c^	0.547	0.032
g_*[Ruminococcus] gnavus* group	2.58^d,e^	3.92^a^	3.57^a,b^	3.57^a,b^	2.01^c^	3.52^a,b^	3.37^b^	3.11^b^	2.87^b^	2.06^b^	0.217	0.001
g_*[Ruminococcus] torques* group	2.69^ef^	4.04^a,b^	3.94^a,b,c^	4.36^a^	3.02^d,ef^	2.28^f^	3.22^c,d,e^	3.23^c,d,e^	3.51^b,c,d^	3.49^b,c,d^	0.266	0.046

sd: standard deviation.

Letters before bacterial groups designate taxa: p_: phylum; c_: class; o_: order; f_: family; g_: genus.

a–f Values in the same row that share a common superscript are not statistically different (*P* > 0.05).

Males presented higher counts of family Muribaculaceae and genus [*Eubacterium*] *brachy* group (*P* < 0.05) and tended to have enrichment of family Eggerthellaceae, and genera *Peptococcus* and *Ruminococcaceae* UCG-014 (*P* < 0.1). Contrarily, female feces tended to have higher counts of Firmicutes and a higher abundance of genus *Fusicatenibacter*.

### Quantitative real-time PCR

The results of the qPCR are displayed in Table 6. Age increased the number of DNA copies of total bacteria, *Clostridium* cluster I, *Enterococci* spp.*, Faecalibacterium prausnitzii* and *Lactobacillus* spp. (*P* < 0.001). The number of DNA copies of *E. coli* was higher at week 28 of age and similar among the remaining weeks (*P* = 0.001). Organic selenium increased the DNA concentration of *Lactobacillus* spp. (*P* = 0.024) and tended to decrease the DNA concentration of *E. coli* (*P* = 0.055). The interaction between selenium source and age tended to affect the number of DNA copies of *Bifidobacterium* spp. (*P* = 0.099; data not shown). *Bifidobacterium* spp. was also affected by gender (*P* = 0.002), being higher in males.

**Table 6.** Log10 copies of bacterial genomic DNA of total bacteria and selected bacterial groups per g of fresh feces of puppies from 20 to 52 weeks of age fed the inorganic (SeInorg) and the organic (SeOrg) selenium supplemented diet.

	Age (weeks)							Selenium source				Gender			
	20	28	36	44	52	SEM	*P* value	SeInorg	SeOrg	SEM	*P* value	F	M	SEM	*P* value
Total bacteria	8.92^b^	10.1^a^	9.85^a^	10.1^a^	10.1^a^	0.164	<0.001	9.79	9.84	0.117	0.607	9.37	9.89	0.131	0.336
*Clostridium* cluster I	5.63^c^	6.58^b^	6.80^b^	6.86^b^	7.42^a^	0.238	<0.001	6.81	6.50	0.187	0.143	6.55	6.77	0.221	0.505
*Escherichia coli*	7.51^a,b^	8.13^a^	7.44^b^	7.30^b,c^	7.07^c^	0.245	0.001	7.58	7.40	0.172	0.055	7.64	7.34	0.204	0.213
*Enterococci* spp.	6.17^b^	7.24^a^	7.43^a^	7.17^a^	7.35^a^	0.170	<0.001	6.99	7.15	0.109	0.141	7.05	7.09	0.119	0.789
*Faecalibacterium prausnitzii*	5.16^c^	6.47^b^	7.71^a^	7.70^a^	7.63^a^	0.180	<0.001	6.97	6.90	0.142	0.328	6.84	7.02	0.142	0.713
*Lactobacillus* spp.	5.55^b^	7.87^a^	7.73^a^	7.58^a^	7.68^a^	0.310	<0.001	7.09	7.47	0.309	0.024	7.17	7.39	0.184	0.182
*Bifidobacterium* spp.^†^	5.48^b^	7.25^a^	6.92^a^	7.01^a^	7.30^a^	0.212	<0.001	6.65	6.94	0.140	0.143	6.53	7.06	0.129	0.002

SEM: standard error of the mean.

Letters from gender designate: F: female; M: male.

a–c Values in the same row that share a common superscript are not statistically different (*P* > 0.05).

† Interaction between selenium source and age tended to be significant (*P* < 0.1).

### Ammonia-N, pH, biogenic amines, lactate and volatile fatty acids

Table 7 presents the pH and the ammonia-N, biogenic amines, lactate and VFA contents of fresh feces collected at five time-points from 20 to 52 weeks of age, and Table   8 displays the interaction between selenium source and age (for *P* < 0.05). Fecal pH and ammonia-N were unaffected by gender (*P* > 0.05), but they were affected by the interaction between selenium source and age (*P* < 0.05). The fecal pH of dogs fed both selenium sources was similar within weeks, except for 44 weeks of age in which SeOrg promoted higher pH than that of SeInorg. Ammonia-N content was the highest at 36 weeks of age for both selenium sources and the lowest at weeks 20 and 52 in feces of dogs fed SeInorg and at weeks 28 and 52 in those fed SeOrg.

**Table 7.** Fecal pH and concentration of end-fermentation products (ammonia, mg/g; biogenic amines, µmol/L; lactate, mM; and volatile fatty acids, VFA, µmol/g) of fresh feces from puppies from 20 to 52 weeks of age fed the inorganic (SeInorg) and the organic (SeOrg) selenium supplemented diets.

	Age (weeks)							Selenium source				Gender			
	20	28	36	44	52	SEM	*P* value	SeInorg	SeOrg	SEM	*P* value	F	M	SEM	*P* value
pH*	6.30^c^	6.30^c^	6.29^c^	6.58^b^	6.71^a^	0.101	<0.001	6.43	6.44	0.075	0.902	6.42	6.45	0.071	0.814
Ammonia-N*	1.18^b,c^	1.22^b,c^	1.76^a^	1.38^b^	1.03^c^	0.126	<0.001	1.28	1.37	0.112	0.497	1.31	1.34	0.120	0.863
Putrescine	2451^a,b^	3406^a^	3317^a^	2545^b^	1811^b^	319.8	<0.001	2553	2859	205.9	0.201	3234	2178	205.9	<0.001
Cadaverine	2343^a,b^	2248^a,b^	2704^a^	2469^a,b^	1522^b^	395.6	0.002	2516	1998	302.7	0.100	3030	1485	302.7	<0.001
Spermidine*	464	417	367	410	379	28.6	0.112	429	386	17.0	0.076	405	409	14.7	0.822
Spermine	361	315	248	261	242	28.2	0.055	298	272	17.6	0.276	283	288	17.6	0.820
Lactate^†^	8.26^a^	3.31^b^	5.09^a,b^	1.61^c^	1.74^c^	0.870	0.001	3.25	4.75	0.676	0.084	4.28	3.73	0.614	0.385
Total VFA	199^a^	178^a^	176^a^	114^b^	126^b^	9.7	<0.001	155	164	5.9	<0.001	162	157	7.1	0.550
Acetate	122^a^	97.3^b^	102^a,b^	58.5^c^	61.7^c^	3.21	<0.001	87.7	89.0	2.80	0.690	91.5	85.2	2.82	0.060
Propionate	46.5^a^	45.5^a^	46.7^a^	30.4^b^	31.8^b^	2.65	<0.001	36.8	43.5	2.83	<0.001	41.4	38.9	3.66	0.586
Butyrate	18.3^b,c^	27.1^a^	19.9^b^	15.5^c^	21.7^a,b^	3.16	<0.001	18.0	23.1	2.37	0.001	21.5	19.6	2.95	0.615
*Iso*-butyrate	2.71	2.42	2.92	2.66	2.70	0.308	0.356	2.89	2.48	0.222	<0.001	2.86	2.51	0.259	0.203
*Iso*-valerate^†^	5.82	4.03	4.90	3.73	3.75	0.430	0.107	4.43	4.46	0.335	0.931	4.90	4.00	0.329	0.020
Valerate**	2.01^a,b^	0.80^c^	0.81^c^	1.84^b^	2.77^a^	0.228	<0.001	1.51	1.79	0.202	0.334	1.69	1.60	0.163	0.582
*Iso*-caproate*	0.61^b^	0.62^b^	0.66^b^	1.02^a^	1.22^a^	0.187	0.002	0.82	0.84	0.188	0.916	0.56	1.10	0.202	0.038
Caproate	1.39^a^	0.55^b^	0.54^b,c^	0.42^c^	0.36^d^	0.058	<0.001	0.65	0.65	0.039	0.722	0.70	0.60	0.040	<0.001
Heptanoate^†^	0.12^a^	0.06^b^	0.06^b^	0.05^b^	0.06^b^	0.009	<0.001	0.07	0.07	0.005	0.995	0.07	0.07	0.005	0.574

SEM: standard error of the mean.

Letters from gender designate: F: female; M: male.

a–c Values in the same row that share a common superscript are not statistically different (*P* > 0.05).

**interaction between selenium source and age was highly statistically significant (*P* < 0.001), *interaction between selenium source and age was statistically significant (*P* < 0.05), ^†^interaction between selenium source and age tended to be significant (*P* < 0.1).

**Table 8.** Fecal pH and concentration of end-fermentation products (ammonia, mg/g; spermidine µmol/L; valerate and *iso*-caproate, µmol/g) as affected (*P* < 0.05) by the interaction between selenium source (inorganic, SeInorg; organic, SeOrg) and age (20–52 weeks).

	SeInorg					SeOrg						
	20	28	36	44	52	20	28	36	44	52	SEM	*P* value
pH	6.4^b,c^	6.2^c^	6.3^c^	6.4^b,c^	6.8^a^	6.2^c^	6.4^b,c^	6.3^c^	6.7^a^	6.6^a,b^	0.13	0.042
Ammonia-N	1.00^c^	1.19^b,c^	1.63^a^	1.16^b,c^	0.83^c^	1.16^b,c^	1.06^c^	1.66^a^	1.39^a,b^	1.03^c^	0.144	0.047
Spermidine	500^a^	451^a^	402^a,b^	475^a^	318^b^	428^a,b^	383^a,b^	332^b^	345^a,b^	441^a^	40.7	0.001
Valerate	2.53^a,b^	0.87^c^	0.78^c^	1.12^c^	2.24^b^	1.50^b,c^	0.73^c^	0.84^c^	2.56^a,b^	3.31^a^	0.323	<0.001
*Iso*-caproate	0.74^a,b,c^	0.78^a,b,c^	0.66^c^	0.69^b,c^	1.22^a,b^	0.48^c^	0.47^c^	0.67^c^	1.36^a^	1.22^a,b^	0.199	0.004

SEM: standard error of the mean.

a–d Values in the same row that share a common superscript are not statistically different (*P* > 0.05).

Contents of fecal putrescine and cadaverine were affected by age (non-patterned variation; *P* < 0.05) and by gender (*P* < 0.001), being higher in females, but not affected by selenium source (*P* > 0.05). The interaction between selenium source and age affected spermidine concentration (*P* = 0.001). Spermidine content was higher at 28 weeks of age in feces of dogs fed SeInorg compared to those fed SeOrg, the opposite being observed at week 52 in which feces of dogs fed SeOrg had higher spermidine than of dogs fed SeInorg.

The total VFA production and concentrations of acetate, propionate, caproate and lactate decreased with age (*P* < 0.001). In turn, *iso*-caproate increased with age (*P* = 0.014), whereas butyrate and valerate fluctuated along the weeks (*P* < 0.05). Organic selenium increased total VFA production and concentrations of butyrate and propionate (*P* = 0.05) and tended to increase lactate concentration (*P* = 0.084). The interaction between selenium source and age affected the concentrations of valerate and *iso*-caproate (*P* < 0.05) and tended to affect lactate, *iso*-valerate and heptanoate (*P* < 0.1, data not shown). Valerate was similar in both selenium sources at weeks 20, 28 and 36, whereas at weeks 40 and 52 was higher in dogs fed SeOrg. Similarly, fecal *iso-*caproate concentration was similar between SeInorg and SeOrg along age, except at week 44, being higher in feces of dogs fed SeOrg. Males had a higher fecal concentration of *iso*-valerate, *iso*-caproate and caproate (*P* < 0.05), and tended to have a higher content of acetate (*P* = 0.060).

## DISCUSSION

This study was designed to evaluate the effects of supplemental selenium source (sodium selenite and selenium-enriched yeast) on the gut microbiome of puppies from 20 to 52 weeks of age. The effects of gender were also evaluated.

Puppies were healthy throughout the length of the study, with no clinical signs of disease and exhibiting normal blood biochemical and hematological parameters.

The results of 16S rRNA gene sequencing generally agree with earlier reports of healthy individuals. Firmicutes was the most abundant phylum, followed by Bacteroidetes, Fusobacteria and Proteobacteria. Previous studies have shown that Firmicutes, Bacteroidetes, Proteobacteria, Fusobacteria, and Actinobacteria were the most abundant phyla in the dogs’ gut microbiome (Beloshapka *et al*. 2013). Among them, *Clostridium* spp., Lactobacillales and Proteobacteria predominate in the small intestine and Clostridiales, *Bacteroides, Prevotella* 9 and Fusobacteria in the large intestine (Suchodolski 2016).

### Effects of age

Alpha diversity metrics evaluate the community richness and evenness within each sample, whereas beta diversity metrics assess the similarity of the community within groups (Lozupone *et al*. 2007). Our results showed that age significantly affected the alpha and beta diversity indexes, with the most notable changes between the 20^th^ week of age and the remaining. In a study performed in dogs from 2 to 56 days of age, beta diversity reached relative stability after 42 days of age, yet differences between dams and puppies were still apparent by the 56^th^ day of age (Guard *et al*. 2017). No study of the gut microbiome of puppies in later stages of development is available. However, a study performed with kittens, observed that the structural and functional diversity of microbiome differ between 18 and 30 weeks of age, but not between 30 and 42 weeks of age (Deusch *et al*. 2015), similarly to our results.

Immediately after birth, the sterile neonatal gastrointestinal tract is colonized by bacteria from the birth canal and the surrounding environment (Buddington 2003). Along with the increase in the number of microorganisms, further growth-related changes involve shifts in the relative abundances of several bacterial groups (Moon *et al*. 2018). Proteobacteria are dominant members of the neonatal gut, which is abundant in oxygen immediately post-partum. They consume oxygen and lower the redox potential of the gut environment, facilitating the proliferation of groups of anaerobic bacteria, allowing them to eventually supplant the aerotolerant forms and dominate the populations of bacteria (Buddington 2003). The post-natal changes in terms of digestive and absorptive capacity of nutrients and the development of enteric immune functions are believed to influence the gut microbiome (Buddington 2003; Moon *et al*. 2018). Therefore, the most significant changes associated with age are expected to occur in the first days to weeks of life. However, we did not evaluate the gut colonization, as the study began when dogs were 12 weeks of age, but instead, we accompanied a later stage of development up to 1-year-old, which allowed us to observe changes in the gut microbiome of dogs during growth.

It is possible that the gut microbiome modulation observed may be related to the physical–anatomical modifications of the host during growth and also to environmental factors. During the length of the trial (8 months), all dogs were subjected to the same housing and husbandry, and thus, they underwent the same environment modifications. One significant change with the potential to alter the community was the introduction and intensification of leash-walks outside the facilities around the 20^th^ week of age. This enabled contact with a microbial-enriched environment. Another meaningful change, not growth- or husbandry-related, might be the season, as the trial was performed during spring, summer and autumn. Although changes in dog's husbandry might have occurred, e.g. shortening of leash-walks in rainy or during heat waves, care was taken to avoid substantial modifications. However, to what extent the seasons may have contributed to a significant change in the gut microbiome of the dogs is a matter that requires further investigation.

Results of qPCR showed an increase in total bacteria with age, agreeing with the results of the selected bacterial groups. The profile of the community suffered modifications with age, as shown by changes in the relative abundance of taxa, and the concentration of bacterial DNA increased with growth, reflecting the incremental gut harboring.

Most notably, a decrease in relative abundance of Firmicutes and an increase in that of Bacteroidetes were observed. A shift on Firmicutes: Bacteroidetes ratio with age was reported in humans (Mariat *et al*. 2009). Bacteroidetes degrade complex carbohydrates into acetate and propionate, whereas Firmicutes are secondary fermenters that further produce butyrate from acetate (Minamoto *et al*. 2019). However, within phyla, there are species able to degrade other nutrients and to produce VFA through different pathways (Rios-Covian *et al*. 2016). In addition to bacterial production and degradation, the decrease of VFA could also be explained by an increase in the absorption of VFA in the colon, thus lowering its fecal excretion (Middelbos, Fastinger and Fahey 2007). Indeed, the upscaling of digestion and absorption capacity of puppies accompanies their growth (Kuzmuk *et al*. 2005).

Biogenic amines, in which polyamines are included, are low molecular weight organic compounds sourced externally in pet food or raw ingredients (Learey *et al*. 2018) or internally by e.g. intestinal microbiota, pancreatic-biliary secretions and dead intestinal cells (Ramos-Molina *et al*. 2019). Exogenous polyamines in foods are usually absorbed before they reach the large bowel (Ramos-Molina *et al*. 2019), so it is likely that an important share found in feces was synthesized in the gut by decarboxylase-positive microorganisms such as Enterobacteriaceae, *Enterococcus, E. coli* and lactic acid bacteria (Espinosa-Pesqueira, Roig-Sagues and Hernandez-Herrero 2018). Putrescine, spermine and spermidine derive from l-arginine or l-ornithine depending on the microorganism involved, and the degradation and recycling of biogenic amines comprise, among others, the conversion of putrescine into spermine and this into spermidine and vice-versa (Fernandez-Reina, Urdiales and Sanchez-Jimenez 2018). These biogenic amines have physiological roles as cell viability, proliferation and correct differentiation (Fernandez-Reina, Urdiales and Sanchez-Jimenez 2018). Cadaverine is formed via lysine decarboxylase (Barbieri *et al*. 2019) and has mitigated the pathogenicity process of *Shigella* spp. (from which is released), due to the protective effect cadaverine can exert on intestinal mucosa from enterotoxins (Tofalo, Cocchi and Suzzi 2019). However, being associated with cell proliferation, polyamines are essential for both normal and neoplastic cells, and indeed, higher levels have been associated with carcinogenesis in dogs (Rossi *et al*. 2015). Putrescine and cadaverine decreased from 20 to 52 weeks of age but had peaks at the 28^th^ and 36^th^ week of age. These observations might be associated with the variation of relative abundance of order Bacilli as these biogenic amines are mostly produced by Gram-negative bacteria (Pugin *et al*. 2017). Also, spermine content tended to decrease with age, which might be due to lower amino acid decarboxylation by bacteria or be related to the decrease of putrescine, a precursor of spermine. It is likely that at a younger age, the requirements for biogenic amines are higher due to growth and the decrease of their content thereafter seems positive for longevity, as their excess is detrimental and relates to tumors and deleterious effects of aging (Matsumoto *et al*. 2011).

### Effects of selenium source

Although the alpha and beta diversity of the community remained unaffected by the selenium source, there were differences in taxa abundance of particular genera and families.

The impact of selenium deficiency on the dog's gut microbiome has not been reported yet, but in mice, it was linked to impairment of gut barrier function and immune responses (Zhai *et al*. 2019). However, the mechanisms by which selenium modulates intestinal bacteria are complex and potentially interlinked. Lv *et al*. (2015) suggested that the antioxidant role of selenium helps to mitigate the diarrhea incidence rate and therefore contributed to a stable and healthier gastrointestinal ecosystem of weanling piglets. This was supported by another study in which selenium supplementation was efficient in controlling intestinal inflammation in rats with induced small intestinal mucositis (Qiu *et al*. 2019). Furthermore, it was also suggested that the positive effects of selenium on the intestinal barrier function and immune system were due to the promotion of beneficial bacteria in rats (Zhai *et al*. 2018).

Considering the source of supplemental selenium, we observed that feces of dogs fed SeOrg tended to have a lower concentration of DNA copies of *E. coli* and a higher DNA concentration of *Lactobacillus*. *E. coli* is harbored by healthy dogs’ intestinal microflora, though it was also associated with gastroenteritis, in the presence of bacterial virulence factors and compromised local or systemic immunity (Marks *et al*. 2011). In broilers, dietary supplementation with inorganic and bacterial organic selenium reduced the number of *E. coli* (Dalia *et al*. 2018), when compared to diets without selenium supplementation. Similarly, piglets fed selenium-enriched probiotics had lower *E. coli* and higher *Lactobacillus* spp. than those fed non-supplemented diets or diets supplemented with sodium selenite (Lv *et al*. 2015). The concentration of lactate tended to be higher in feces of dogs fed SeOrg, agreeing with the significantly higher DNA concentration of *Lactobacillus*. Selenium can promote the growth and activity of lactic acid bacteria that are capable of incorporating selenium from the growth media (Arauz *et al*. 2008), and these bacteria might inhibit pathogenic microorganisms through secretion of hydrogen peroxide, acids and other antimicrobial substances (Dalia *et al*. 2018).

Even though SeInorg diet promoted the enrichment of VFA producers, namely genera *Catenibacterium, Holdemanella* and *Ruminococcaceae* UCG-014, we observed higher total production of VFA and higher concentrations of propionate and butyrate in feces of dogs fed SeOrg. It is important to note that the relative abundance of those genera was < 0.5%, which can contribute to explain the lack of correlation between them and the concentration of fermentation products. Volatile fatty acids are sources of energy for the enterocytes and have immunomodulatory properties, thus being essential for the health of the host's gut (Suchodolski 2016). Therefore, by promoting a higher concentration of VFA, the organic source of selenium appears to be advantageous for supplementation over inorganic selenium.

Our results suggest that the selenium sources under test modulated differently the gut microbiome. This observation agreed with a study of Dalia *et al*. (2018), which compared the combination of vitamin E with either sodium selenite or bacterial organic selenium for broiler supplementation, observing an increase of DNA concentrations of *Lactobacilli, Bifidobacteria* and a decrease of *E. coli* and *Salmonella*.

### Interaction between selenium source and age

Taxa abundance and concentration of some end-fermentation products were affected by the interaction between age and source of selenium.

Decrease in the abundance of phylum Actinobacteria and class Coriobacteriia during growth was more pronounced in puppies fed SeInorg diet. Studies have demonstrated that supplementation with fructooligosaccharides and galactooligosaccharides increased the relative abundance of phylum Actinobacteria in cats (Barry *et al*. 2012) and humans (Davis *et al*. 2011), respectively. Members of Coriobacteriia, affected by the interaction of age and selenium, included families Eggerthellaceae and Coriobacteriaceae (genus *Collinsella*), involved in steroid and bile salt metabolism, which is particularly relevant to ameliorate the consequences of metabolic diseases (Clavel *et al*. 2014). In humans, *Collinsella* has been associated with poor metabolic states, increased level of cholesterol and LDL in healthy adults, and cardiovascular diseases (Frost *et al*. 2019). Although no correlation of this bacteria with dog metabolic disease was reported, the use of probiotics seems to help control the growth of *Collinsella* (Xu *et al*. 2019).

Abundance of genus *Allisonella* (Firmicutes) presented a higher fluctuation with age in dogs fed the SeOrg diet, with the lowest value being found at week 20 of age. In humans, a decrease in *Allinsonella* was associated with irritable bowel syndrome associated with predominant constipation or diarrhea (Hills *et al*. 2019), though reports in dogs are not available. Genus *Dubosiella* (Firmicutes), which was found to be lower in dogs fed SeInorg at 52 weeks of age, is poorly documented in the dog's gut.

In dogs fed SeInorg, [*Ruminococcus*] *gnavus* group and [*Ruminococcus*] *torques* group (Firmicutes) showed a higher fluctuation during growth of puppies than those fed the SeOrg diet. A relation between selenium supplementation and *Ruminococcus* was not yet reported in dogs. In humans, [*Ruminococcus*] *torques*, a butyrate-producing bacteria, is associated with anti-inflammatory activity and was found diminished in Crohn's disease, a chronic immune-mediated inflammatory condition (Maldonado-Contreras *et al*. 2020). However, in dogs, it was positively associated with inflammatory cytokine interleukin-6 and tumor necrosis factor-alpha (Xu *et al*. 2019).

The decrease in the abundance of Lachnospiraceae family (Firmicutes) was more pronounced in puppies fed the SeInorg diet. A reduction of Lachnospiraceae, important VFA producers, has been associated with inflammatory bowel disease, supporting its role in the maintenance of gastrointestinal health (Suchodolski *et al*. 2012).

Fecal pH during growth were differently affected by selenium source, being more constant with SeInorg. Changes in pH can affect microbial communities, thus impacting the concentration and profile of fermentation products (Ilhan *et al*. 2017; Henrick *et al*. 2018).

Ammonia-N content in feces results from cumulative effects of enterocyte metabolism and degradation of peptides and amino acids in the gut (Diether and Willing 2019). According to a study performed in humans, the enrichment in bacteria from the *Clostridium* genus, and species of *Enterococcus, Shigella* and *E. coli*, were correlated with an increase of ammonia-N (Richardson, McKain and Wallace 2013). However, in our study, no correlation was found between ammonia-N content and *Clostridium* genus abundance, which may be partly explained by the higher use of dietary protein as energy source by dogs than humans (Romsos and Ferguson 1983).

The concentration of spermidine was similar in feces of dogs fed inorganic and organic selenium up to the 44^th^ week of age, while at week 52 feces from dogs fed SeOrg registered higher concentration of spermidine. As differences were only detected in one week, the interaction effect should be interpreted with caution. Increased spermidine content was associated with low protein digestibility in the study reported by Pinna *et al*. (2016). In the present study, the SeOrg diet presented slightly lower dietary protein levels and digestibility than SeInorg (data not shown), which might support an effect of amino acid availability for degradation.

*In vitro* studies showed valerate to inhibit the growth of *Clostridioides difficile* (McDonald *et al*. 2018), an important enteropathogen, which would have been interesting to quantify in the present study. Moreover, valerate can modulate the immune response, controlling Th17‐mediated responses induced by segmented filamentous bacteria (Luu and Visekruna 2019). We observed the highest fecal valerate at week 52 in dogs fed SeOrg, which might suggest a benefit of the supplementation with organic selenium.

In turn, fecal *iso-*caproate was similar between selenium sources within weeks, except at week 44, in which a higher concentration was observed in feces of dogs fed SeOrg. *Iso-*caproate is a minor branched-chain fatty acid formed through oxidation of leucine yet poorly documented in terms of biological effects. *C. difficile* was reported to produce it from the degradation of l-leucine, by first oxidizing the amino acid with the formation of *iso*-valerate and later reduction (in presence of CO_2_) to *iso*-caproate (Kim *et al*. 2006). In neonatal humans, it has been positively correlated with Xanthomonadaceae (Proteobacteria) and *Staphylococcus* (Firmicutes) and negatively correlated with *Bifidobacterium* (Del Chierico *et al*. 2015). However, in our study, both DNA concentration and counts of *Bifidobacterium* were not affected by the supplemental selenium source.

### Effect of gender

Gender did not affect the alpha and beta diversity of the gut microbiome, yet we observed differences in counts of a few bacterial groups. Genus *Fusicatenibacter* was higher in females, which agrees with a result reported in healthy humans (Hirakawa *et al*. 2019). In males, [*Eubacterium*] *brachy* group and family Muribaculaceae were more abundant. Despite in dogs the gender effect has not been reported in these taxa, a higher abundance of Muribaculaceae was reported in male wild type mice (Son *et al*. 2019).

The metabolites of protein degradation (e.g. putrescine, cadaverine, caproate and *iso*-valerate) were higher in females. The variation of these metabolites and concentration of VFA can be related to shifts in colon microbial composition, but also with changes in the content or digestibility of diet protein, or in the host digestive/absorptive capacity of peptides/amino acids in the small intestine (Neis, Dejong and Rensen 2015). We were not able to attribute these results to the microbiome since bacteria associated with colonic proteolysis, e.g. *Bacteroides, Clostridium, Fusobacterium, Lactobacillus* and *Streptococcus* (Hoyles and Swann 2019) were not higher in females. However, we observed that the digestibility of crude protein was lower in females (data not shown), which appears to correlate with the increase of these metabolites.

In addition to differences in taxa abundance, qPCR revealed a higher number of DNA copies of *Bifidobacterium* in males. In a study performed in growing kittens, sexual development did not affect the microbiome (Deusch *et al*. 2015). Also, in dogs, no gender-related differences were reported (Jha *et al*. 2020). Nevertheless, the dissimilarities in immune processes driven by sex hormones and sex-linked immune response genes, already described in the literature, could potentialy affect gut microbiota (Vemuri *et al*. 2019) of puppies.

In the present study, the gender effects should be interpreted with caution due to the limted number of animals used. Nevertheless, our innovative findings highlight the importance of conducting further research to understand sex-driven differences in the gut microbiome of dogs.

## CONCLUSION

The gut microbiome of puppies shifted with growth. We observed differences in both alpha and beta diversity, and an overall increase in the relative abundance of Bacteroidetes and a decrease of Firmicutes. Moreover, DNA concentration of total bacteria and selected bacterial groups increased with age, while the fecal total VFA production and concentrations of butyrate, propionate and acetate decreased, which might be explained by increased absorption. Gender had a minor effect on microbiome composition, affecting only some individual taxa and a few fecal end-fermentation products. Although selenium source did not affect alpha and beta diversity, it modulated the gut microbiome of dogs differently. Organic selenium tended to decrease the DNA concentration of *E. coli*, an important enteropathogen, and increased that of *Lactobacillus*. However, the effects of selenium source on gut microbiome relative abundance may be affected by growth. Total VFA, butyrate and propionate concentrations were promoted by organic selenium, which is beneficial for the gut immunity and health of puppies.

## Supplementary Material

fiaa212_Supplemental_Files
