# Supplementary material for: Supplemental selenium source on gut health: insights on fecal microbiome and fermentation products of growing puppies
Source: FEMS Microbiol Ecol. 2020 Oct 12;96(11):fiaa212. doi: 10.1093/femsec/fiaa212 (PMC7580910; doi:10.1093/femsec/fiaa212)
Supplement: fiaa212_Supplemental_Files [file fiaa212_supplemental_files.zip › Supplementary_Fig_S1_S2_S3.docx]

**Rarefaction Curves**

| 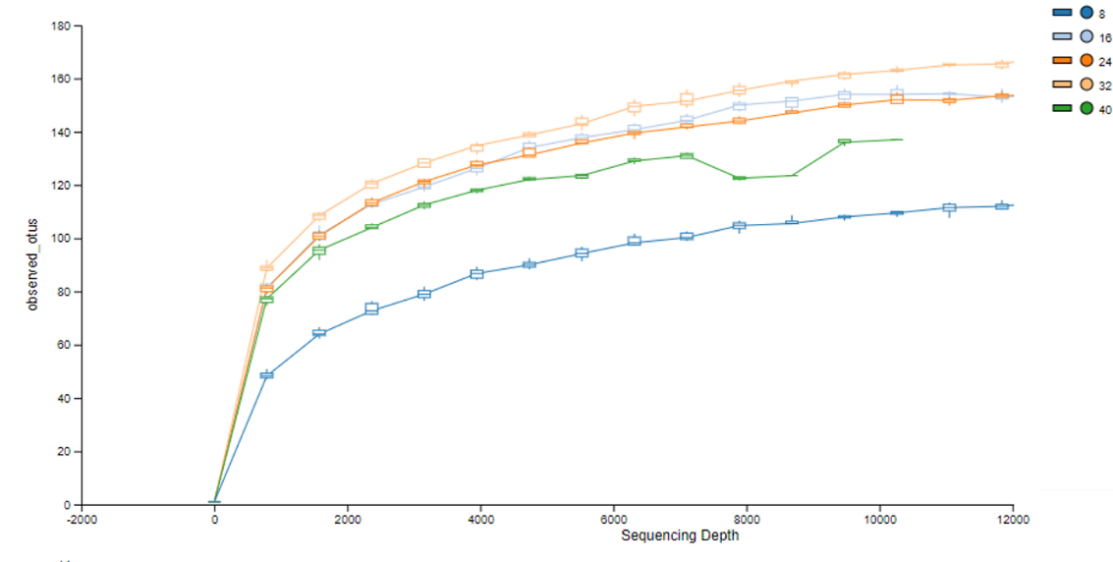**A** |
| --- |
| 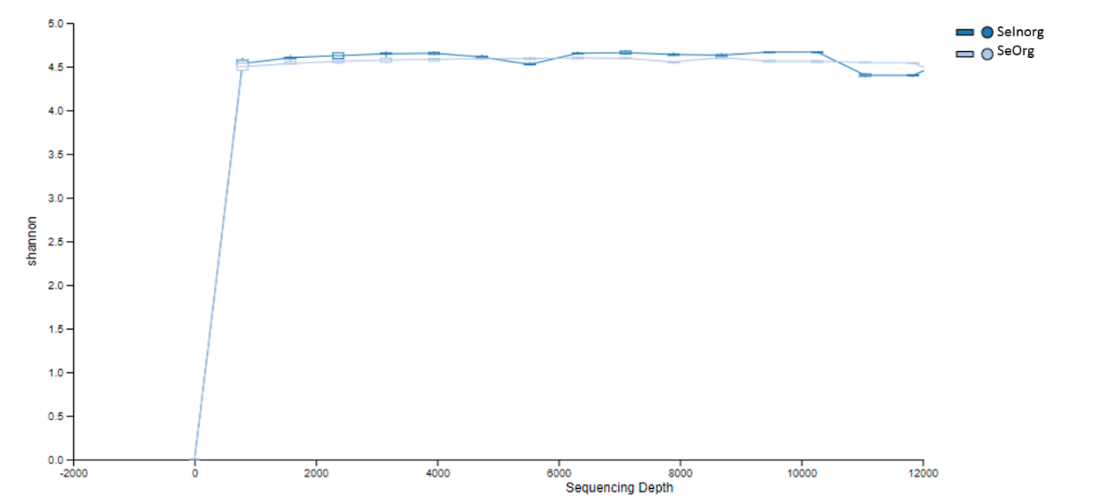**B** |
| 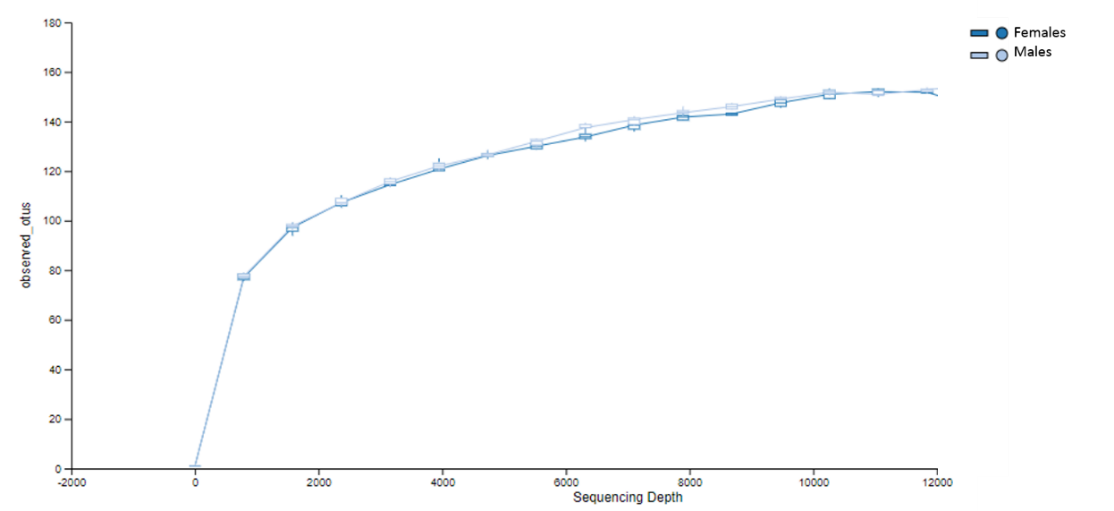**C** |

**Fig. S1*.*** Observed OTU’s: rarefaction curves of communities in feces of dogs according to categories, (A) age, (B) selenium source, and (C) gender

| **A**  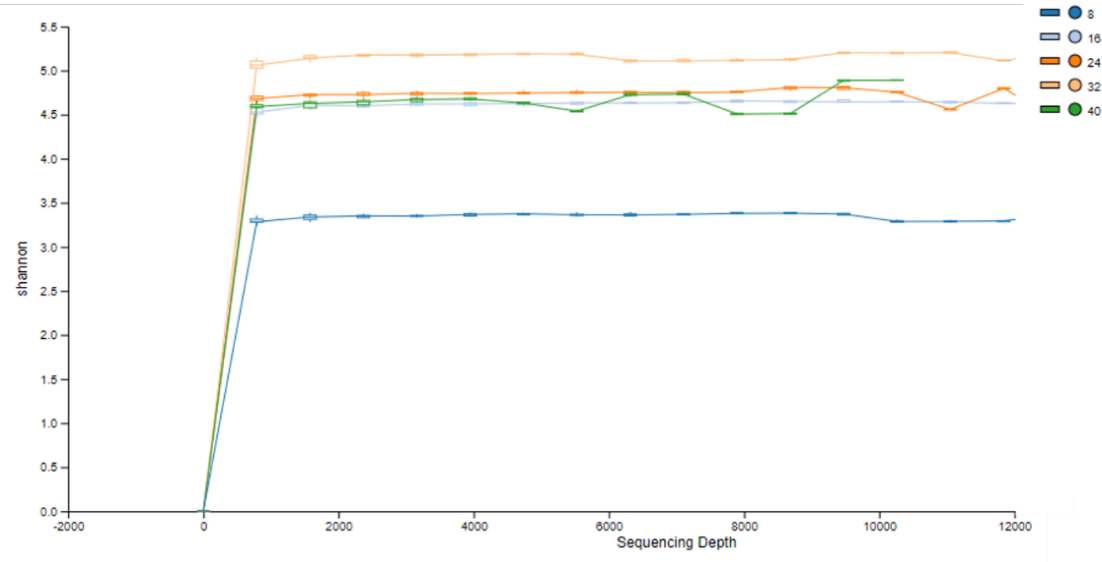 |
| --- |
| **B**  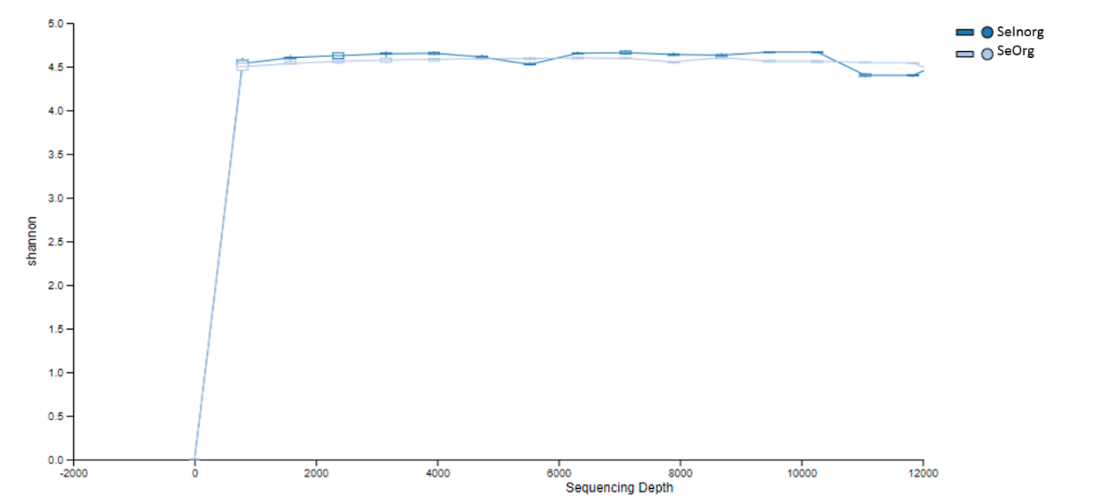 |
| **C**  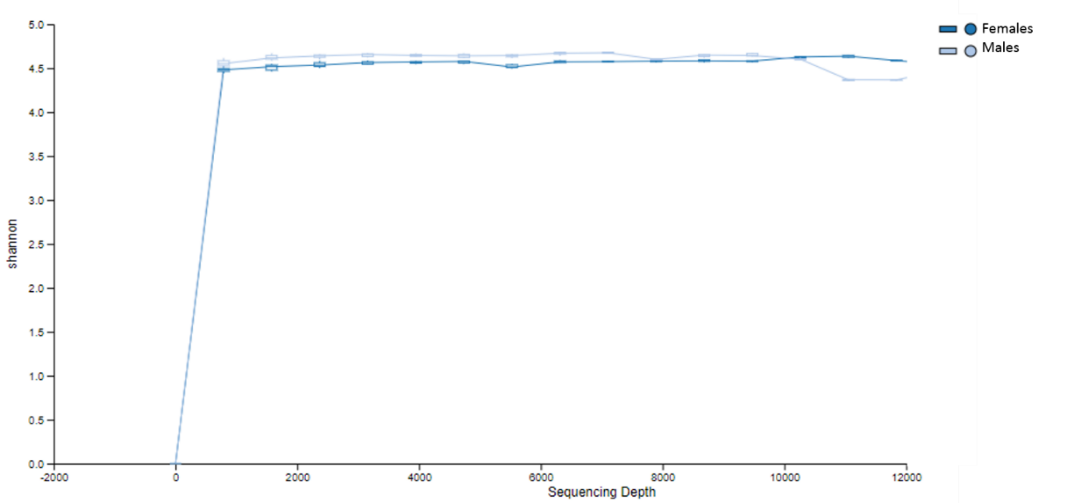  **Fig. S2.** Shannon’s diversity index: rarefaction curves of communities in feces of dogs according to categories, (A) age, (B) selenium source, and (C) gender |

| 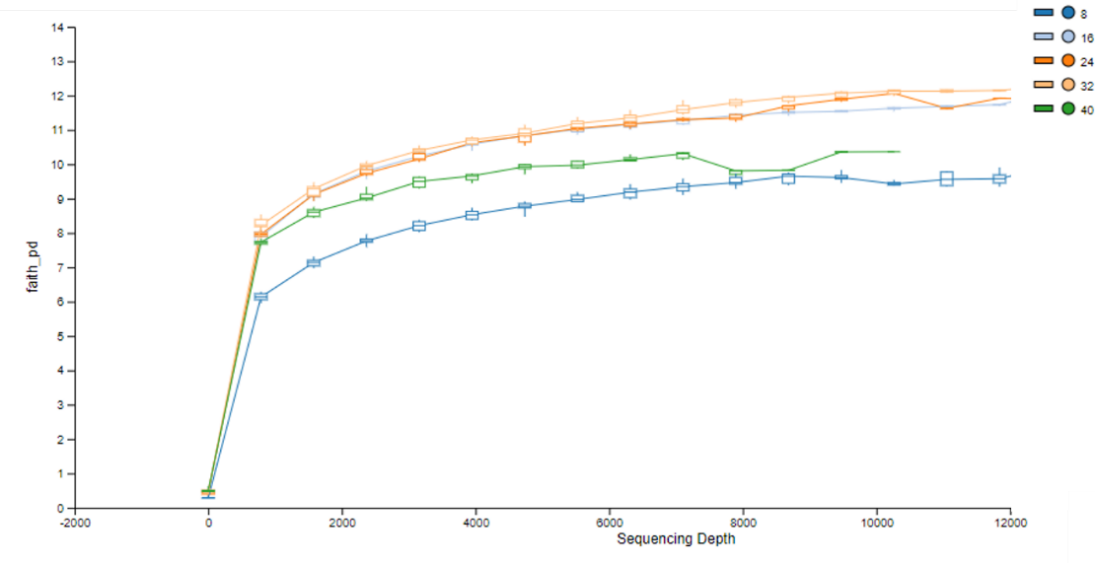**A** |
| --- |
| 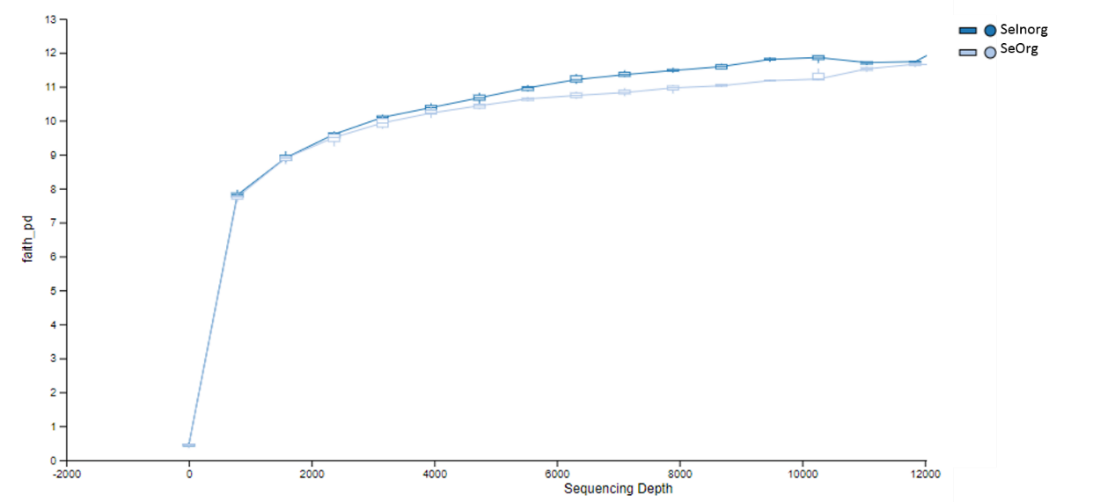**B** |
| 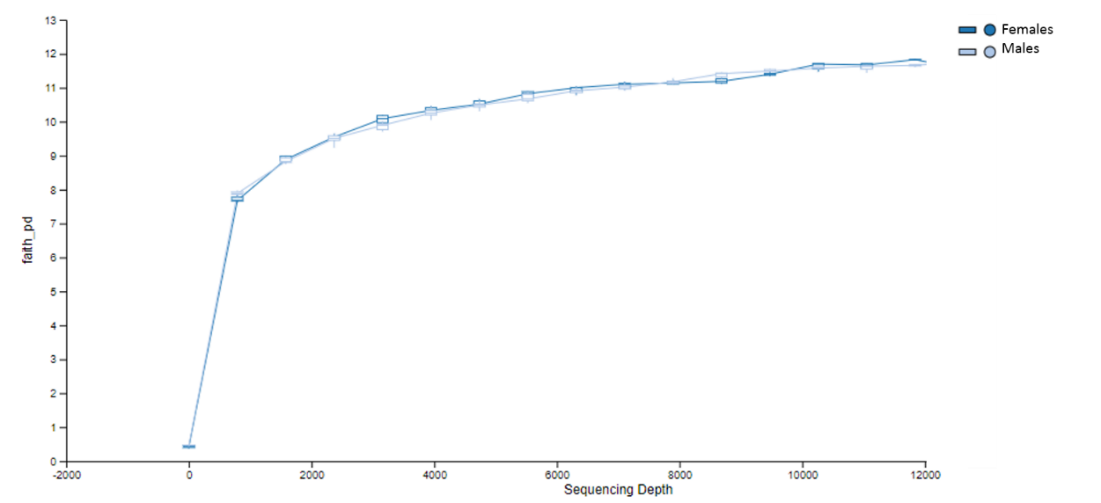**C** |

**Fig. S3*.*** Faith’ Phylogenetic diversity: rarefaction curves of communities in feces of dogs according to categories, (A) age, (B) selenium source, and (C) gender
