# Supplementary material for: Supplemental selenium source on gut health: insights on fecal microbiome and fermentation products of growing puppies
Source: FEMS Microbiol Ecol. 2020 Oct 12;96(11):fiaa212. doi: 10.1093/femsec/fiaa212 (PMC7580910; doi:10.1093/femsec/fiaa212)
Supplement: fiaa212_Supplemental_Files [file fiaa212_supplemental_files.zip › Supplementary_Table_S1_S2_S3_S4.docx]

**Additional file 3**

**Table S1.** Kruskal-Wallis pairwise comparison of alpha diversity metrics of the fecal microbiome of puppies during growth

| Metrics for age (weeks) | | H | P-value | *q*-value |
| --- | --- | --- | --- | --- |
| Shannon’s diversity index | | | | |
| 28 (n=12) | 36 (n=12) | 0.00 | 1.000 | 1.000 |
| 28 (n=12) | 44 (n=12) | 2.08 | 0.149 | 0.237 |
| 28 (n=12) | 52 (n=10) | 0.73 | 0.391 | 0.476 |
| 28 (n=12) | 20 (n=12) | 15.4 | 0.000 | 0.001 |
| 36 (n=12) | 44 (n=12) | 1.92 | 0.166 | 0.237 |
| 36 (n=12) | 52 (n=10) | 0.63 | 0.429 | 0.476 |
| 36 (n=12) | 20 (n=12) | 13.7 | 0.000 | 0.001 |
| 44 (n=12) | 52 (n=10) | 2.94 | 0.086 | 0.173 |
| 44 (n=12) | 20 (n=12) | 13.7 | 0.000 | 0.001 |
| 52 (n=10) | 20 (n=12) | 12.2 | 0.000 | 0.001 |
| Faith's phylogenetic diversity | | | | |
| 28 (n=12) | 36 (n=12) | 0.56 | 0.453 | 0.503 |
| 28 (n=12) | 44 (n=12) | 0.03 | 0.862 | 0.862 |
| 28 (n=12) | 52 (n=10) | 5.63 | 0.018 | 0.035 |
| 28 (n=12) | 20 (n=12) | 11.2 | 0.001 | 0.008 |
| 36 (n=12) | 44 (n=12) | 0.65 | 0.419 | 0.503 |
| 36 (n=12) | 52 (n=10) | 5.33 | 0.021 | 0.035 |
| 36 (n=12) | 20 (n=12) | 8.33 | 0.004 | 0.013 |
| 44 (n=12) | 52 (n=10) | 5.33 | 0.021 | 0.035 |
| 44 (n=12) | 20 (n=12) | 9.72 | 0.002 | 0.009 |
| 52 (n=10) | 20 (n=12) | 3.41 | 0.065 | 0.093 |
| Pielou's Evenness | | | | |
| 28 (n=12) | 36 (n=12) | 0.00 | 0.954 | 0.954 |
| 28 (n=12) | 44 (n=12) | 2.25 | 0.133 | 0.222 |
| 28 (n=12) | 52 (n=10) | 0.07 | 0.792 | 0.880 |
| 28 (n=12) | 20 (n=12) | 14.5 | 0.000 | 0.001 |
| 36 (n=12) | 44 (n=12) | 1.33 | 0.248 | 0.355 |
| 36 (n=12) | 52 (n=10) | 0.21 | 0.644 | 0.805 |
| 36 (n=12) | 20 (n=12) | 12.8 | 0.000 | 0.001 |
| 44 (n=12) | 52 (n=10) | 2.72 | 0.099 | 0.199 |
| 44 (n=12) | 20 (n=12) | 14.5 | 0.000 | 0.001 |
| 52 (n=10) | 20 (n=12) | 12.2 | 0.000 | 0.001 |

**Table S2.** Kruskal-Wallis pairwise comparison of alpha diversity metrics of the fecal microbiome of puppies fed diets supplemented with inorganic (SeInorg) or organic (SeOrg) selenium

| Metrics for diet | | H | P-value | *q*-value |
| --- | --- | --- | --- | --- |
| Shannon’s diversity index | | | | |
| SeInorg | SeOrg | 0.23 | 0.630 | 0.630 |
| Faith's phylogenetic diversity | | | | |
| SeInorg | SeOrg | 1.59 | 0.208 | 0.208 |
| Pielou's Evenness | | | | |
| SeInorg | SeOrg | 0.11 | 0.744 | 0.744 |

**Table S3.** Kruskal-Wallis pairwise comparison of alpha diversity metrics of the fecal microbiome of puppies according to gender

| Metrics for gender | | H | P-value | *q*-value |
| --- | --- | --- | --- | --- |
| Shannon’s diversity index | | | | |
| Female | Male | 0.04 | 0.846 | 0.846 |
| Faith's phylogenetic diversity | | | | |
| Female | Male | 0.20 | 0.658 | 0.658 |
| Pielou's Evenness | | | | |
| Female | Male | 0.002 | 0.969 | 0.969 |

**Table S4.** Permutation multivariate analysis PERMANOVA pairwise on Unweighted and Weighted UniFrac distances in fecal microbiome of dogs according to age

| Beta diversity metrics for age (weeks) | | Sample size | Pseudo-F | P-value | *q-*value |
| --- | --- | --- | --- | --- | --- |
| Unweighted UniFrac distances | | | | | |
| 28 | 36 | 24 | 2.14 | 0.005 | 0.006 |
| 28 | 44 | 24 | 2.70 | 0.001 | 0.001 |
| 28 | 52 | 22 | 3.11 | 0.001 | 0.001 |
| 36 | 44 | 24 | 2.27 | 0.006 | 0.007 |
| 36 | 52 | 22 | 3.52 | 0.001 | 0.001 |
| 44 | 52 | 22 | 2.08 | 0.008 | 0.008 |
| 20 | 28 | 24 | 7.95 | 0.001 | 0.001 |
| 20 | 36 | 24 | 7.91 | 0.001 | 0.001 |
| 20 | 44 | 24 | 8.92 | 0.001 | 0.001 |
| 20 | 52 | 22 | 9.30 | 0.001 | 0.001 |
| Weighted UniFrac distances | | | | | |
| 28 | 36 | 24 | 1.07 | 0.351 | 0.351 |
| 28 | 44 | 24 | 1.13 | 0.341 | 0.351 |
| 28 | 52 | 22 | 2.30 | 0.066 | 0.110 |
| 36 | 44 | 24 | 1.16 | 0.311 | 0.351 |
| 36 | 52 | 22 | 3.13 | 0.044 | 0.088 |
| 44 | 52 | 22 | 2.00 | 0.119 | 0.170 |
| 20 | 28 | 24 | 16.6 | 0.001 | 0.003 |
| 20 | 36 | 24 | 10.2 | 0.001 | 0.003 |
| 20 | 44 | 24 | 15.7 | 0.001 | 0.003 |
| 20 | 52 | 22 | 20.3 | 0.001 | 0.003 |
